# Supplementary material for: The Signaling Pathways Project, an integrated ‘omics knowledgebase for mammalian cellular signaling pathways
Source: Sci Data. 2019 Oct 31;6:252. doi: 10.1038/s41597-019-0193-4 (PMC6823428; doi:10.1038/s41597-019-0193-4)
Supplement: Supplementary file 1 — Supplementary information [file 41597_2019_193_MOESM1_ESM.pdf]

The Signaling Pathways Project, an integrated ‘omics knowledgebase for mammalian cellular signaling pathways

Scott Ochsner, David Abraham, Kirt Martin, Wei Ding, Apollo McOwiti, Wasula Kankanamge, Zichen Wang, Kaitlyn Andreano, Ross Hamilton, Yue Chen, Angelica Hamilton, Marin Gantner, Michael Dehart, Shijing Qu, Susan Hilsenbeck, Lauren Becnel, Dave Bridges, Avi Maayan, Janice Huss, Fabio Stossi, Charles Foulds, Anastasia Kralli, Donald McDonnell & Neil J. McKenna

SUPPLEMENTARY INFORMATION

**Section 1**	SPP UI walk-through……………………………………………....	Page 2
	1A. Datasets………………………………………………………..	Page 3
	1B. Ominer single gene target queries………………………………..	Page 5
	1C. Ominer Gene Ontology term queries………………………….........	Page 7
	1D. Ominer custom gene set upload……...…………………………...	Page 8
	1E. Consensomes………………………………………………...........	Page 9
**Section 2**	Full list of transcriptomic consensomes in initial release of SPP	Page 12
**Section 3**	Literature and ChIP-Seq cross-validation of top-ranked transcriptomic consensome targets………………………………....	Page 22
**Section 4**	Gene targets in the 99th percentile of the mouse All nodes liver transcriptomic consensome………………………………………	Page 26
**Section 5**	Genes encoding metabolic enzymes in the 99th percentile of the All nodes Mm liver transcriptomic consensome, deficiency of whose human orthologs is associated with a metabolic disorder………………………………………………………………	Page 40
**Section 6**	Bench validation use case 2: ERR family members and insulin receptor regulate targets encoding glycogen synthase phosphatase and kinase regulatory subunits……………………	Page 43
**Section 7**	Bench validation use case 3: the murine ERR, PPARGC and adipose tissue consensomes implicate *Mcrip2* in adipocyte oxidative metabolism………………………………………………	Page 45
**Section 8**	Transcriptomic consensome calculation for a hypothetical gene	Page 46
**Section 9**	Q-PCR primers for consensome validation……………………...	Page 47

Section 1. SPP UI walk-through

Please note: SPP is a work in progress: our coverage of all signaling pathway nodes represented in GEO or SRA is dictated by the availability of funding for biocurator support and gaps are inevitable. Please send suggestions for curation of datasets for specific nodes to support@signalingpathways.org. Kindly report any bugs (or send feedback) to support@signalingpathways.org.

Annotated datasets can be browsed in the Datasets section of the SPP website (Subsection 1A).

You can ask questions of the SPP knowledgebase in the Ominer search engine (Subsections 1B-1E) which allows users to explore evidence for regulatory relationships between signaling pathway nodes and their downstream genomic targets.

Decision tree indicating SPP user interface options for various user strategies

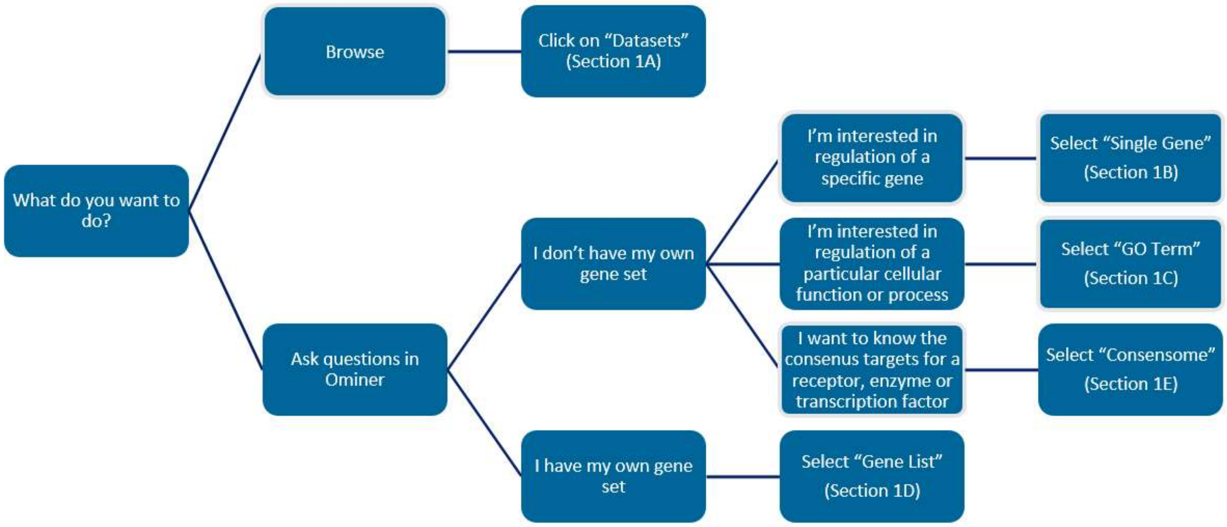

1A. Datasets.

If you simply wish to browse the datasets in SPP, you can do so at https://www.signalingpathways.org/datasets/index.jsf.

You can filter the datasets by signaling pathway module category, biosample, species or ‘omics type.

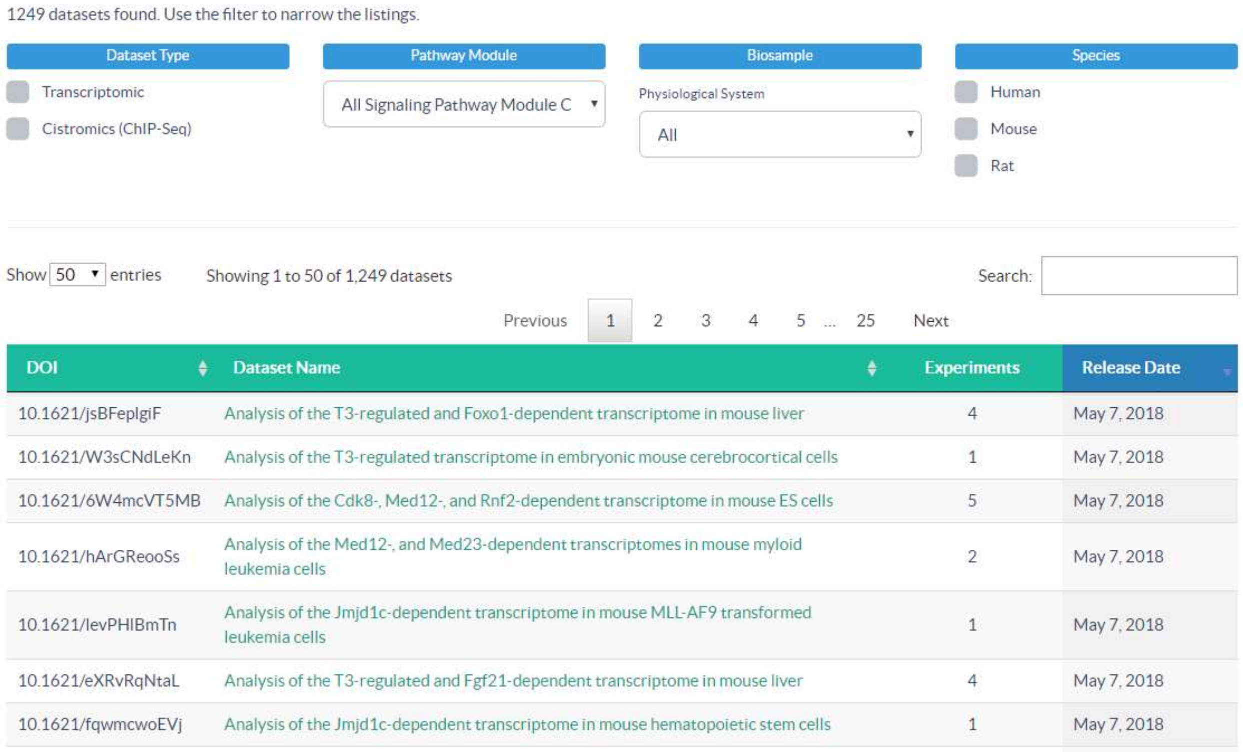

DOI	Dataset Name	Experiments	Release Date
10.1621/jsBFeplgiF	Analysis of the T3-regulated and Foxo1-dependent transcriptome in mouse liver	4	May 7, 2018
10.1621/W3sCNdLeKn	Analysis of the T3-regulated transcriptome in embryonic mouse cerebrocortical cells	1	May 7, 2018
10.1621/6W4mcVT5MB	Analysis of the Cdk8-, Med12-, and Rnf2-dependent transcriptome in mouse ES cells	5	May 7, 2018
10.1621/hArGReooSs	Analysis of the Med12-, and Med23-dependent transcriptomes in mouse myloid leukemia cells	2	May 7, 2018
10.1621/levPHIBmTn	Analysis of the Jmjd1c-dependent transcriptome in mouse MLL-AF9 transformed leukemia cells	1	May 7, 2018
10.1621/eXRvRqNtaL	Analysis of the T3-regulated and Fgf21-dependent transcriptome in mouse liver	4	May 7, 2018
10.1621/fqwmcwoEVj	Analysis of the Jmjd1c-dependent transcriptome in mouse hematopoietic stem cells	1	May 7, 2018

Clicking on the name displays the Dataset page, which displays essential biocurated information, as well as the targets with the highest differential expression values (transcriptomic datasets) or the targets with the highest MACS2 scores (cistromic/ChIP-Seq datasets). Clicking on the target names on the left hand side will run a default single target gene query across the entire SPP knowledgebase.

Analysis of the T3-regulated and Foxo1-dependent transcriptome in mouse liver

Overview

Dataset Name : Analysis of the T3-regulated and Foxo1-dependent transcriptome in mouse liver

Description : Liver tissue was isolated from 8 week old C57BL/6 male mice 3 d after injection with Foxo1 siRNA or control siRNA followed by injection of 10 µg/kg bw T3 or vehicle.

Dataset Type : Transcriptomic

Release Date : May 08, 2018

DOI : 10.1621/jsBFeplgiF

Version : This is Version 1.0 of an annotated derivative of the original dataset, which can be found in GSE68803

Dataset Citation : Yen PM, Singh BK, Tripathi M and Ghosh S (2016) Analysis of the T3-regulated and Foxo1-dependent transcriptome in mouse liver, v1.0. SignalingPathway Project Datasets. 10.1621/jsBFeplgiF

Download Citation :

Associated Article : Singh BK, Sinha RA, Zhou J, Tripathi M, Ohba K, Wang ME, Astapova I, Ghosh S, Hollenberg AN, Gauthier K and Yen PM (2016) Hepatic FOXO1 Target Genes Are Co-regulated by Thyroid Hormone via RICTOR Protein Deacetylation and MTORC2-AKT Protein Inhibition. J. Biol. Chem. **291** 198-214 View Abstract | View PubMed | View Article

Download Dataset

Experiments

There are 3 Experiments for this dataset. Up to the 50 up and down p < 0.05 FDR data points are displayed for each. Please download the dataset to view all data points.

1 | T3 vs Con

Gene List

Scatter Plot | Conditions

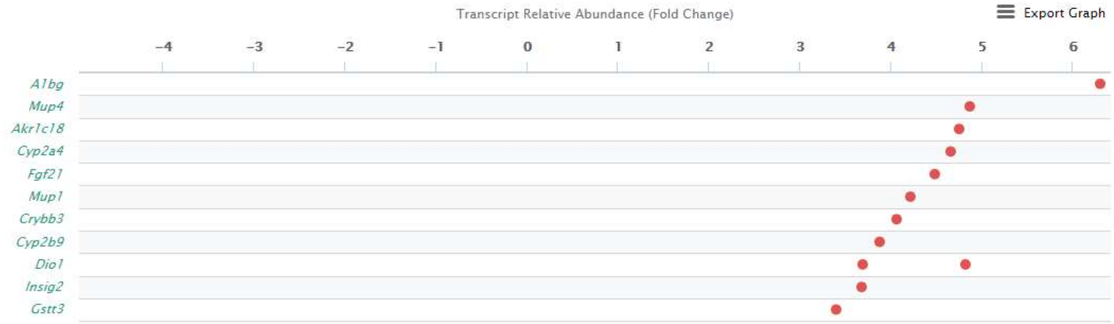

1B. Ominer single gene target queries.

1) In Ominer, select Single Gene from "Target gene(s) of Interest"

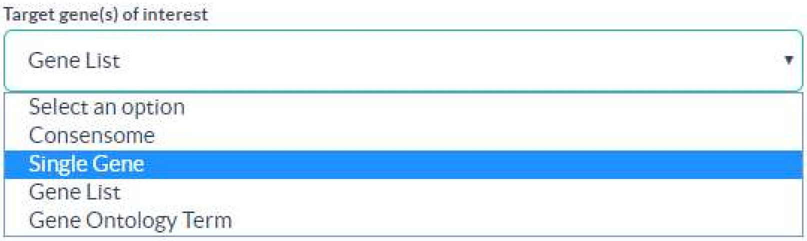

2) Select an 'omics Category

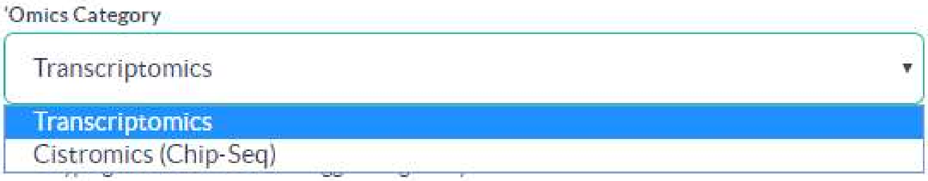

3) Start typing a gene name and select from the drop down suggestions (***you will not be able to submit the query unless you select from the drop down***).

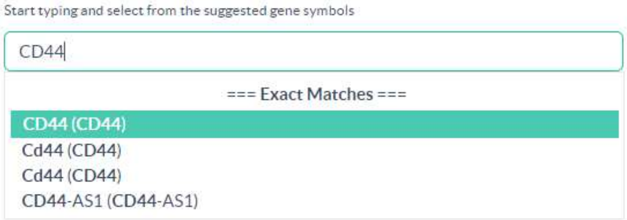

4) Specify a Signaling Pathway Module Category/Class/Family of interest. We recommend the default setting (All) since this returns the most regulatory information for a genomic target.

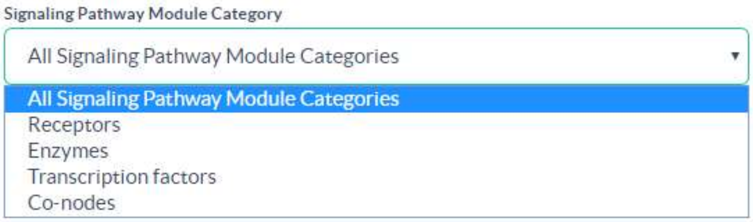

5) Select a Physiological System and/or Organ & species of interest. Again, the default settings are recommended since these will return the most regulatory information for a target of interest.

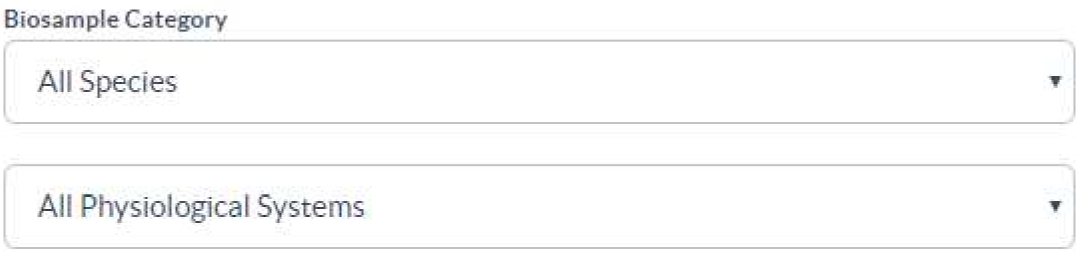

6) Select an FDR Significance Cut-off. We recommend the default (5E-02) unless your query returns more than the maximum of 3,000 data points, in which case the cut-off should be iteratively increased to reduce the number of data points.

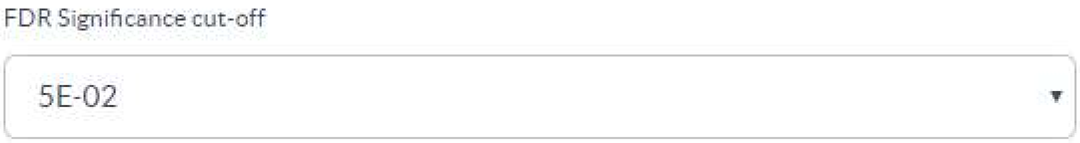

7) Submit your query

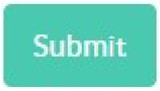

8) SPP will return a **Regulation Report** for your target gene of interest. This is a detailed synopsis of the signaling pathway nodes whose genetic or small molecule manipulation impacts expression of your target of interest (transcriptomic Reports); or the signaling pathway nodes that bind within -/+ 10 kb of the transcription start site of your target gene of interest (cistromic/ChIP-Seq reports). Users can use these Reports to hypothesize candidate signaling pathways regulating their target gene of interest.

The default display is by Target, this can be changed to Pathway Module Category, Biosample or Species using the drop-down in the top left. The layout for transcriptomic and cistromic reports is slightly different so we will briefly summarize each in turn.

TRANSCRIPTOMIC REPORTS

The horizontal axis is the transcript relative abundance (fold change).

The vertical axis indicates the Category, Class and Family of the manipulated node. The bottom level labels indicated any genetic (in italics e.g. overexpression, knockdown) or pharmacological (in bold, e.g. BSM administration) node manipulations.

Note: To facilitate database design, some SPP designated families contain only a single node (e.g. androgen receptor, glucocorticoid receptor).

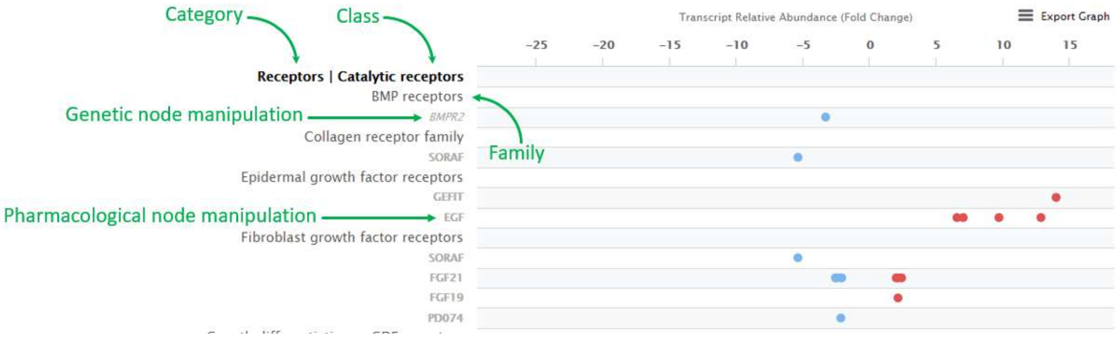

CISTROMIC/CHIP-SEQ REPORTS

The horizontal axis is the MACS2 peak height within -/+ 10 kb of the transcription start site of the target gene of interest. MACS2 values are obtained from the ChIP-Atlas resource (Oki et al, BioRxiv 262899).

The vertical axis indicates the Category, Class and Family of the ChIP-Seq antigen. The bottom level labels indicate, in order, the node antigen (normal font); any BSMs in the experiment design (bold) and any genetic manipulations (e.g. overexpression, knockdown) in italics.

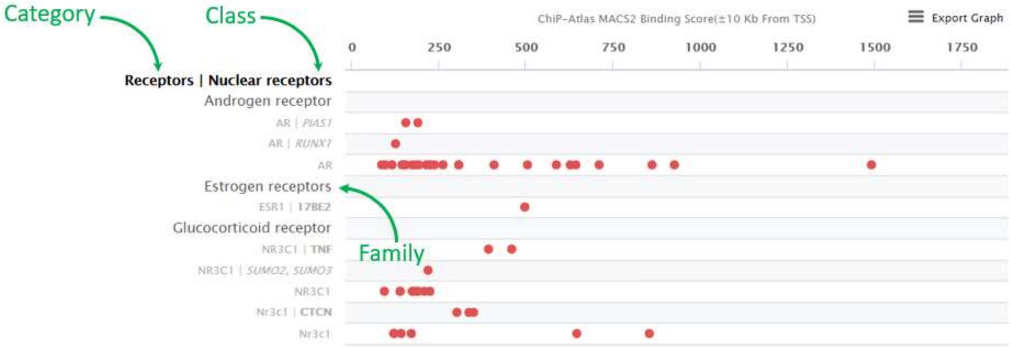

Data points in Regulation Reports are fully interactive. Click on any data point to see its details, and to navigate to a page describing the experiment, as well as a link to the full dataset page.

1C. Ominer Gene Ontology term queries.

1) In Ominer, select “Gene Ontology Term” from “Target Gene(s) of interest”

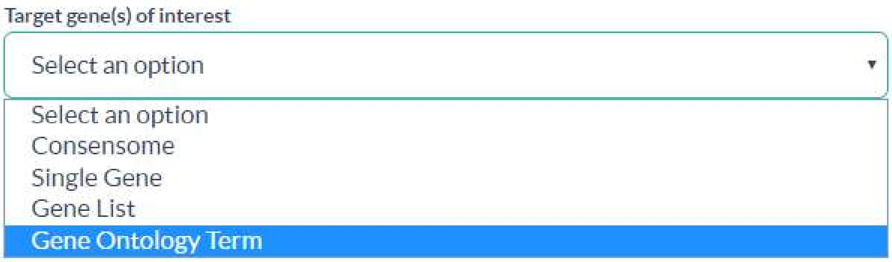

2) Select an ‘omics Category (default is transcriptomic)

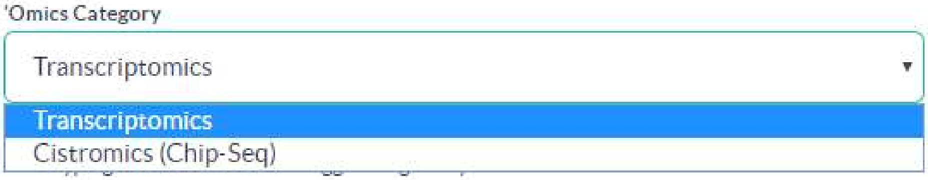

3) Start typing a Gene Ontology term and select from the drop down suggestions (***you will not be able to submit the query unless you select from the drop down***).

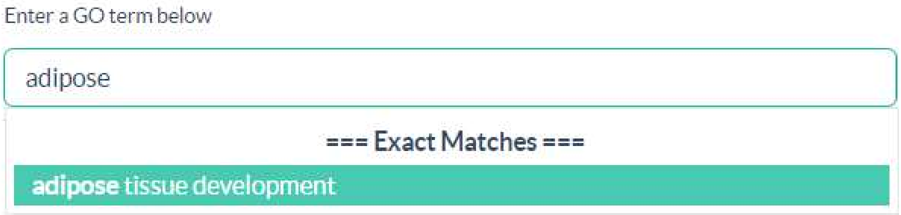

4) To reduce the load on the server, **you <u>must</u> select a Signaling Pathway Module Category (e.g. Enzymes) AND Class (e.g. Kinases) before submitting a GO term query.**

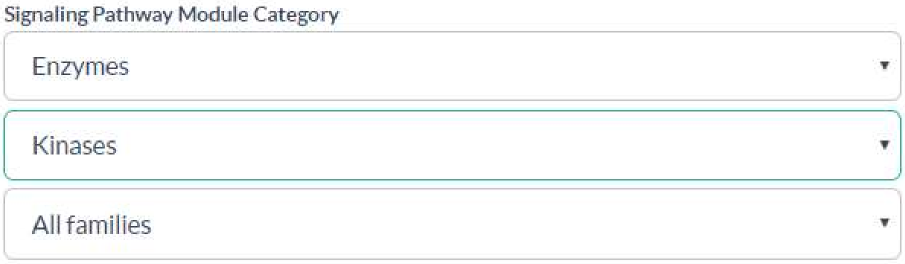

Proceed as in steps 5, 6 and 7 above. The default species is human. As a multi-gene query, the query time may be longer for GO Term queries.

The default display is by Target, this can be changed to Pathway Module Category or Biosample using the drop-down in the top left.

1D. Ominer custom gene set upload

You may upload a custom gene list to evaluate evidence for signaling pathway node regulation of multiple genes of interest. Custom gene lists must

- be in the form of CSV files
- contain approved human, mouse or rat gene symbols or Entrez GeneIDs
- contain no more than 500 records in

Queries returning less than 300 data points will be available for drill-down in the user interface, or for dowload. Queries of more than 3000 data points will be available for download only. Queries returning more than 66,000 data points must be broken down into smaller queries using the query form parameters.

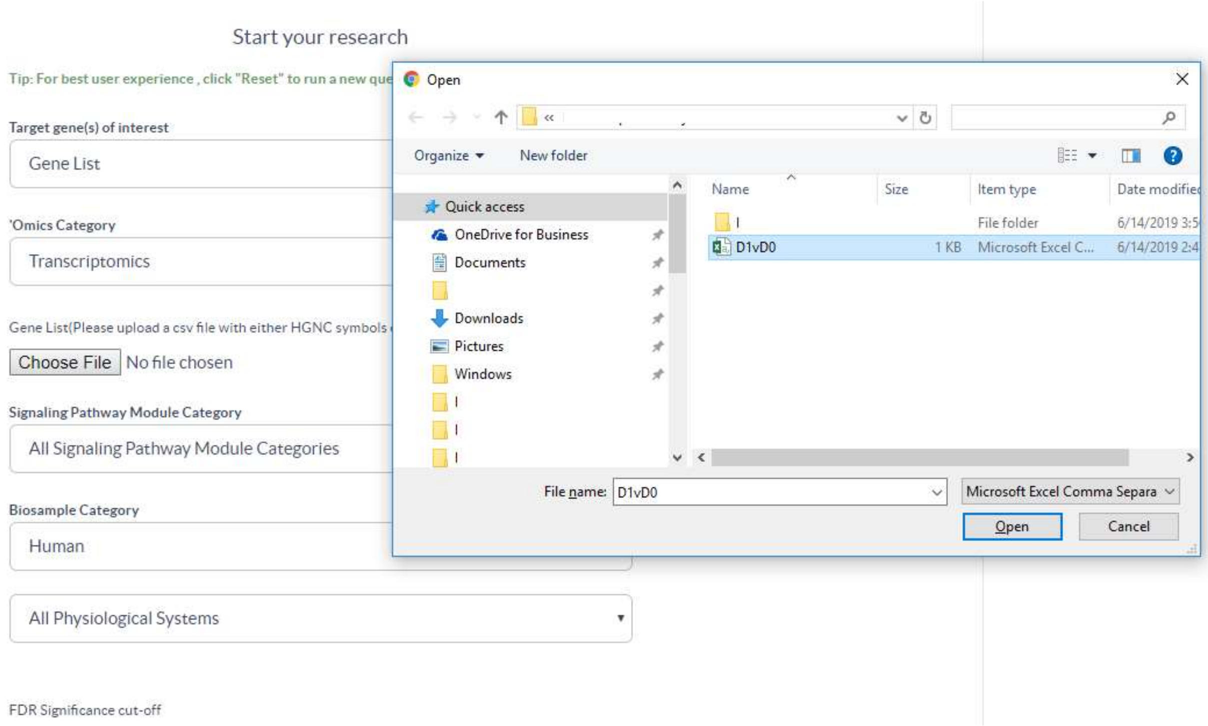

1E. Consensomes.

Consensomic (for consensus 'omics) analysis generates lists called consensomes, in which genes are ranked by frequency of their significant differential expression in response to manipulation of nodes in a given pathway node family (transcriptomic consensomes), or the average MACS2 peak scores for all nodes in a given family (cistromic/ChIP-Seq consensomes).

1) In Ominer, select "Consensome" from the "Target gene(s) of interest"

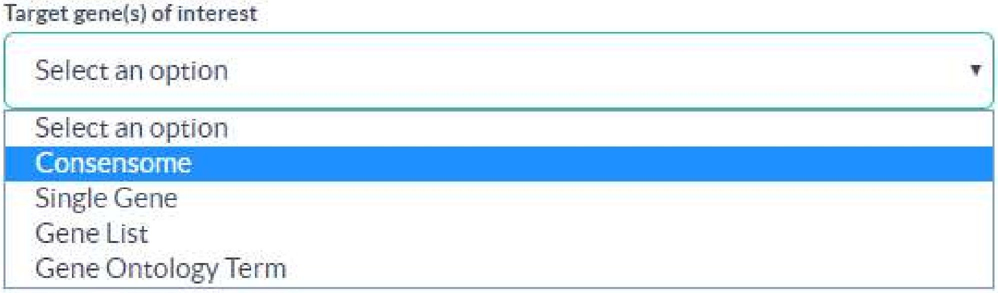

2) Select an ‘omics Category (default is transcriptomic)

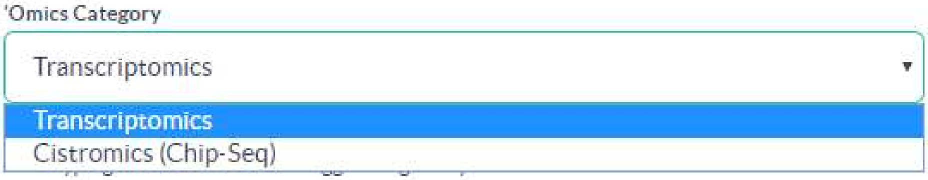

3) Select a Signaling Pathway Module Family,

You must select a Catetgory, Class **and** Family to view a consensome, otherwise the Submit button will not become active.

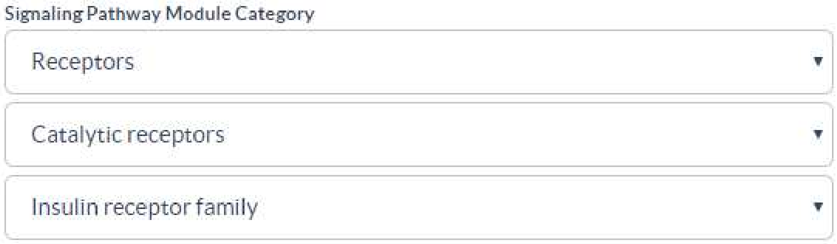

4) Select a Physiological System or Organ. If sufficient curated datasets are available, SPP calculates conensomes for specific Physiological Systems or Organs. If not, only an “All Physiological Systems” consensome will be available.

5) If a consensome is available for your family of interest, the following message will be displayed

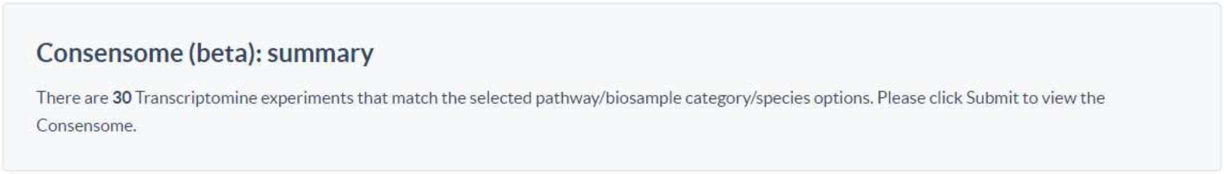

If not, you will see the following message

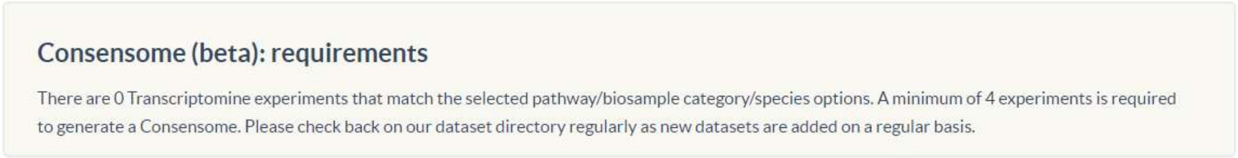

Practical staffing limitations restrict our progress to provide consensomes for all possible node families & omics categories. Some are not currently possible at all due to a shortage of deposited datasets. If you have a suggestion for a node family for which you would like to see a consensome (e.g. Patched receptors), please send an e-mail to support@signalingpathways.org. Feedback like this helps us direct our biocuration resources to best serve the research community.

6) Results

Transcriptomic consensomes are displayed in ascending order of consensome p-value, which is the probability that the observed frequency of differential expression occurred by chance. Gene target names link to a transcriptomic Regulation Report for that target showing the data points underlying the consensome.

For either consensome type, the top 10% of targets are displayed in the browser. The full conensome can be downloaded by clicking “Download Results”.

Consensome (beta)

Download Results

Category: Receptors
Class: Catalytic receptors
Family: Insulin receptor family
Species: Human
Physiological System: All
Organ: All

Consensomes are list of genes ranked according to a meta-analysis of their differential expression in publicly archived transcriptomic datasets involving perturbations of a specific signaling pathway in a given biosample category. Consensome are intended as a guide to identifying those genes most consistently impacted by a given pathway in a given tissue context.

Calculated across **619, 882** data points from **30** experiments in **12** datasets.

Show 50 entries

Search:

Target	Gene Name	Discovery Rate	GMFC	CPV	Percentile
PFKFB3	6-phosphofructo-2-kinase/fructose-2,6-biphosphatase 3	0.913	1.383	1.09E-25	99
GRPEL1	GrpE like 1, mitochondrial	0.913	1.452	1.09E-25	99
GEM	GTP binding protein overexpressed in skeletal muscle	0.87	1.963	1.46E-23	99
CCNG2	cyclin G2	0.87	1.996	1.46E-23	99
ARC	activity regulated cytoskeleton associated protein	0.87	1.619	1.46E-23	99
AVPI1	arginine vasopressin induced 1	0.87	1.384	1.46E-23	99
IER3	immediate early response 3	0.87	1.418	1.46E-23	99
YRDC	yrdC N6-threonylcarbamoyltransferase domain containing	0.87	1.682	1.46E-23	99
PMAIP1	phorbol-12-myristate-13-acetate-induced protein 1	0.87	2.015	1.46E-23	99

Cistromic consensomes are displayed in descending order of MACS2 peak value for all nodes in a given target family. Gene target names link to a cistromic/ChIP-Seq Regulation Report for that target showing the data points underlying the consensome.

Section 2. Full list of transcriptomic consensomes in initial release of SPP.

Type	DOI	Family	Physiological System	Organ	Species
Transcriptomic	10.1621/L7ryO4nfGT.1	Abl family kinases (ABL)	ALL	ALL	Human
Transcriptomic	10.1621/OUaswUDREo.1	Adrenergic receptors	ALL	ALL	House Mouse
Transcriptomic	10.1621/xZXYSIb66a.1	Ahr-like	Metabolic	Liver	House Mouse
Transcriptomic	10.1621/uvIOTxojSB.1	Ahr-like	Metabolic	ALL	House Mouse
Transcriptomic	10.1621/71JfGxZa88.1	Ahr-like	ALL	ALL	House Mouse
Transcriptomic	10.1621/CIZ76GPaIZ.1	AIRE	Immune	Thymus	House Mouse
Transcriptomic	10.1621/6sFBg4bPMX.1	AIRE	Immune	ALL	House Mouse
Transcriptomic	10.1621/WT2Z848B2M.1	AIRE	ALL	ALL	House Mouse
Transcriptomic	10.1621/dZO9ARWRrA.1	ALL	Cardiovascular	Vasculature	Human
Transcriptomic	10.1621/GdRqIZCpIL.1	ALL	Female reproductive	Mammary gland	Human
Transcriptomic	10.1621/gcZVQLLBRq.1	ALL	Metabolic	Liver	House Mouse
Transcriptomic	10.1621/wAfROXIkqM.1	ALL	Female reproductive	Uterus	Human
Transcriptomic	10.1621/EAyfNha9j6.1	ALL	Female reproductive	Mammary gland	House Mouse
Transcriptomic	10.1621/NKcuOFvrlW.1	ALL	Female reproductive	Uterus	House Mouse
Transcriptomic	10.1621/Il8wdILO5k.1	ALL	Immune	Leukocytes	Human
Transcriptomic	10.1621/V3zF72HO4r.1	ALL	Metabolic	Liver	Norway Rat
Transcriptomic	10.1621/xqg5au6qGj.1	ALL	Neurosensory	CNS	House Mouse
Transcriptomic	10.1621/3HA1NnZXGw.1	ALL	Skeletal	Bone	Human
Transcriptomic	10.1621/ZEQwZVv9tq.1	ALL	Male reproductive	Prostate	Human
Transcriptomic	10.1621/oCt6pq5zrS.1	ALL	Metabolic	Skeletal muscle	House Mouse
Transcriptomic	10.1621/NYokig3Vww.1	ALL	Cardiovascular	Heart	House Mouse
Transcriptomic	10.1621/1IXtkxGZZC.1	ALL	Metabolic	Adipose tissue	Human
Transcriptomic	10.1621/pdaec2oBuX.1	ALL	Male reproductive	Testis	House Mouse
Transcriptomic	10.1621/SZ91lnhg1Z.1	ALL	Other	Stem cells	House Mouse
Transcriptomic	10.1621/RJOspi4wWX.1	ALL	Respiratory	Lung	Human
Transcriptomic	10.1621/csscxT6esb.1	ALL	Metabolic	Skeletal muscle	Norway Rat
Transcriptomic	10.1621/s1raevLHrx.1	ALL	Metabolic	Liver	Human
Transcriptomic	10.1621/ow1SZEwNWc.1	ALL	Metabolic	Adipose tissue	House Mouse

Transcriptomic	10.1621/nUPWxTmEvF.1	ALL	Cardiovascular	Vasculature	House Mouse
Transcriptomic	10.1621/Wtgh4V65Je.1	ALL	Other	Fibroblasts	House Mouse
Transcriptomic	10.1621/17A5vw3NgQ.1	ALL	Other	Skin	Human
Transcriptomic	10.1621/8ZaOGdbiZl.1	ALL	Respiratory	Lung	House Mouse
Transcriptomic	10.1621/Fx6IMA2ZhJ.1	ALL	Gastrointestinal	Colon	Human
Transcriptomic	10.1621/accyhNC7yH.1	ALL	Metabolic	Kidney	Norway Rat
Transcriptomic	10.1621/HKSPU9w7DC.1	ALL	Gastrointestinal	Colon	House Mouse
Transcriptomic	10.1621/ZjcXrmds56.1	ALL	Immune	Leukocytes	House Mouse
Transcriptomic	10.1621/U3ZhpYxJvB.1	ALL	Other	Skin	House Mouse
Transcriptomic	10.1621/9QPq92lmpO.1	ALL	Blood	HSPCs	House Mouse
Transcriptomic	10.1621/DV685hObyg.1	ALL	Immune	Thymus	House Mouse
Transcriptomic	10.1621/r9Fqyi9rGk.1	ALL	Female reproductive	Placenta	House Mouse
Transcriptomic	10.1621/DjjBxnEPOW.1	ALL	Cardiovascular	ALL	Human
Transcriptomic	10.1621/mqp99oLOMH.1	ALL	Female reproductive	ALL	Human
Transcriptomic	10.1621/L55kOnvHgI.1	ALL	Metabolic	ALL	House Mouse
Transcriptomic	10.1621/cysXM63ofu.1	ALL	Female reproductive	ALL	House Mouse
Transcriptomic	10.1621/FZ2YeZAJmw.1	ALL	Female reproductive	ALL	Norway Rat
Transcriptomic	10.1621/KRqIsfPpXg.1	ALL	Immune	ALL	Human
Transcriptomic	10.1621/b14bAiuBZT.1	ALL	Metabolic	ALL	Norway Rat
Transcriptomic	10.1621/UTzRg52LOd.1	ALL	Neurosensory	ALL	House Mouse
Transcriptomic	10.1621/99Y1UCn8Y4.1	ALL	Skeletal	ALL	Human
Transcriptomic	10.1621/m2wPH4Bz3B.1	ALL	Male reproductive	ALL	Human
Transcriptomic	10.1621/pwFsTHQZqC.1	ALL	Gastrointestinal	ALL	House Mouse
Transcriptomic	10.1621/AICZOg6UBW.1	ALL	Cardiovascular	ALL	House Mouse
Transcriptomic	10.1621/tll8Oo8bna.1	ALL	Metabolic	ALL	Human
Transcriptomic	10.1621/MIFFYZwRyz.1	ALL	Male reproductive	ALL	House Mouse
Transcriptomic	10.1621/GnLFc7ct8R.1	ALL	Other	ALL	House Mouse
Transcriptomic	10.1621/FUMgYbHTiy.1	ALL	Respiratory	ALL	Human
Transcriptomic	10.1621/4ygEbjWUaY.1	ALL	Other	ALL	Human
Transcriptomic	10.1621/EiKBKRPXNY.1	ALL	Blood	ALL	Human
Transcriptomic	10.1621/ApbEp3dJa9.1	ALL	Respiratory	ALL	House Mouse
Transcriptomic	10.1621/zSva4CLFsD.1	ALL	Gastrointestinal	ALL	Human
Transcriptomic	10.1621/zpjZMt7X4Y.1	ALL	Immune	ALL	House Mouse

Transcriptomic	10.1621/DktoUCPBA6.1	ALL	Blood	ALL	House Mouse
Transcriptomic	10.1621/lKP3CQi5sd.1	Androgen receptor	Male reproductive	Prostate	Human
Transcriptomic	10.1621/n5BZZY6OwF.1	Androgen receptor	Male reproductive	Testis	House Mouse
Transcriptomic	10.1621/3I6Tnh3vUd.1	Androgen receptor	Male reproductive	ALL	Human
Transcriptomic	10.1621/CHiLQWXWxl.1	Androgen receptor	Male reproductive	ALL	House Mouse
Transcriptomic	10.1621/TxVb4ifVEX.1	Androgen receptor	Metabolic	ALL	House Mouse
Transcriptomic	10.1621/Xuaz4dyQxT.1	Androgen receptor	ALL	ALL	Human
Transcriptomic	10.1621/YjauVBA6hX.1	Androgen receptor	ALL	ALL	House Mouse
Cistromic	10.1621/Ew4bEahypc.1	Androgen receptor	All	All	House Mouse
Cistromic	10.1621/oNV5SeBSRR.1	Androgen receptor	All	All	Human
Cistromic	10.1621/LPfAHbcMFr.1	C/EBP family	All	All	House Mouse
Cistromic	10.1621/zJO3GG4s7t.1	C/EBP family	All	All	Human
Transcriptomic	10.1621/A1rw9CjTAk.1	C-Akt kinases (AKT)	ALL	ALL	House Mouse
Cistromic	10.1621/vPcOZt9MdC.1	CBP/p300	All	All	Human
Cistromic	10.1621/JromglRlyU.1	CBP/p300	All	All	House Mouse
Transcriptomic	10.1621/17Gwficyu3.1	Collagen receptor family	ALL	ALL	Human
Cistromic	10.1621/UxfhTZ64J4.1	CREB-like factors	All	All	House Mouse
Cistromic	10.1621/oNMFhK8x3b.1	CREB-like factors	All	All	Human
Transcriptomic	10.1621/i1Px53udXG.1	Cyclin-dependent kinases (CDK)	Female reproductive	ALL	Human
Transcriptomic	10.1621/gyY3kgh1Es.1	Cyclin-dependent kinases (CDK)	ALL	ALL	Human
Transcriptomic	10.1621/KB3MOFA1eA.1	E2F family	Female reproductive	ALL	House Mouse
Transcriptomic	10.1621/LLAUQrs5JY.1	E2F family	ALL	ALL	House Mouse
Cistromic	10.1621/OB3ZexC1aL.1	E2F family	All	All	House Mouse
Cistromic	10.1621/CfvWEuWICo.1	E2F family	All	All	Human
Transcriptomic	10.1621/zADqgkIXTN.1	Epidermal growth factor receptors	Female reproductive	Mammary gland	Human
Transcriptomic	10.1621/kX52uTHASE.1	Epidermal growth factor receptors	Female reproductive	ALL	Human
Transcriptomic	10.1621/nN8rZd1okG.1	Epidermal growth factor receptors	ALL	ALL	Human
Transcriptomic	10.1621/JaBE1pYhgf.1	Epidermal growth factor receptors	ALL	ALL	House Mouse

Transcriptomic	10.1621/NCKMJxKklE.1	Estrogen receptors	Female reproductive	Mammary gland	Human
Transcriptomic	10.1621/UH745ztFls.1	Estrogen receptors	Female reproductive	Uterus	House Mouse
Transcriptomic	10.1621/TyaTomxmQ8.1	Estrogen receptors	Metabolic	Liver	Norway Rat
Transcriptomic	10.1621/BYRcrphDrO.1	Estrogen receptors	Female reproductive	ALL	Human
Transcriptomic	10.1621/cUbphXKOnS.1	Estrogen receptors	Female reproductive	ALL	House Mouse
Transcriptomic	10.1621/PX6lA4DQcb.1	Estrogen receptors	Female reproductive	ALL	Norway Rat
Transcriptomic	10.1621/VtQFikSj4y.1	Estrogen receptors	Metabolic	ALL	Norway Rat
Transcriptomic	10.1621/aS8hFkyFTe.1	Estrogen receptors	ALL	ALL	Human
Transcriptomic	10.1621/quqGhwj8vn.1	Estrogen receptors	ALL	ALL	Norway Rat
Transcriptomic	10.1621/PJF8NEyazd.1	Estrogen receptors	ALL	ALL	House Mouse
Cistromic	10.1621/2BKfA4znUo.1	Estrogen receptors	All	All	Human
Cistromic	10.1621/BJ7kkEFKgd.1	Estrogen receptors	All	All	House Mouse
Transcriptomic	10.1621/72axf8m391.1	Estrogen-related receptors	ALL	ALL	Human
Transcriptomic	10.1621/AMjW8Lrezy.1	Estrogen-related receptors	ALL	ALL	House Mouse
Cistromic	10.1621/FR3UZluQd2.1	Estrogen-related receptors	All	All	Human
Cistromic	10.1621/Ff7Lo3xZNJ.1	Estrogen-related receptors	All	All	House Mouse
Cistromic	10.1621/iFskvhHhDn.1	Ets-like	All	All	Human
Cistromic	10.1621/WjJlPOInd2.1	Ets-like	All	All	House Mouse
Transcriptomic	10.1621/SmZO1t1TgH.1	Farnesoid X receptor (FXR)	Metabolic	Liver	House Mouse
Transcriptomic	10.1621/jeDVjnfZKl.1	Farnesoid X receptor (FXR)	Metabolic	ALL	House Mouse
Transcriptomic	10.1621/KJBzdZDiD4.1	Farnesoid X receptor (FXR)	ALL	ALL	House Mouse
Transcriptomic	10.1621/emxBKA5xT7.1	Fibroblast growth factor receptors	ALL	ALL	Human
Transcriptomic	10.1621/NzDv9cvgJY.1	FOXA family	Male reproductive	Prostate	Human
Transcriptomic	10.1621/ZseuiSq6xE.1	FOXA family	Male reproductive	ALL	Human
Transcriptomic	10.1621/jzETFZJh44.1	FOXA family	ALL	ALL	Human
Cistromic	10.1621/zUMBX1AanX.1	FOXA family	All	All	House Mouse
Cistromic	10.1621/IPik89l2Ux.1	FOXA family	All	All	Human
Cistromic	10.1621/8vyVFJI94o.1	FOXC family	All	All	Human
Cistromic	10.1621/yXMhK2rrCV.1	FOXD family	All	All	Human
Cistromic	10.1621/3IpbhsUOUo.1	FOXF family	All	All	House Mouse
Cistromic	10.1621/iBQBrWw3Ft.1	FOXF family	All	All	Human
Cistromic	10.1621/BHD95AnIk3.1	FOXH family	All	All	Human
Cistromic	10.1621/uWfo918s4U.1	FOXJ family	All	All	Human
Cistromic	10.1621/UtyAUmJ2xd.1	FOXK family	All	All	Human

Cistromic	10.1621/B98u6ho8WE.1	FOXK family	All	All	House Mouse
Cistromic	10.1621/dZ8lzlMKhr.1	FOXL family	All	All	House Mouse
Cistromic	10.1621/dGon3b5t5k.1	FOXM family	All	All	Human
Cistromic	10.1621/hQbAQkObkl.1	FOXO family	All	All	Human
Cistromic	10.1621/G1VUbsoJR7.1	FOXO family	All	All	House Mouse
Cistromic	10.1621/WEaOphxlDz.1	FOXP family	All	All	House Mouse
Cistromic	10.1621/bnkUfJR17j.1	FOXP family	All	All	Human
Cistromic	10.1621/edB3TBW9yZ.1	FOXQ family	All	All	Human
Cistromic	10.1621/7RQJp4O72F.1	FOXR family	All	All	Human
Transcriptomic	10.1621/PfUD47GTR6.1	Free fatty acid receptors	Immune	Leukocytes	Human
Transcriptomic	10.1621/sBgJwXnHX5.1	Free fatty acid receptors	Metabolic	Adipose tissue	House Mouse
Transcriptomic	10.1621/nAabU8cSjR.1	Free fatty acid receptors	Immune	ALL	Human
Transcriptomic	10.1621/kyH5SN36wG.1	Free fatty acid receptors	Metabolic	ALL	House Mouse
Transcriptomic	10.1621/Y1aJckvDIO.1	Free fatty acid receptors	ALL	ALL	House Mouse
Transcriptomic	10.1621/bdnZTAbCHG.1	Free fatty acid receptors	ALL	ALL	Human
Transcriptomic	10.1621/RUTfCxDO5H.1	G protein-coupled estrogen receptor	Female reproductive	Mammary gland	Human
Transcriptomic	10.1621/jixZkdOn1G.1	G protein-coupled estrogen receptor	Female reproductive	Uterus	House Mouse
Transcriptomic	10.1621/1QsHjWYjms.1	G protein-coupled estrogen receptor	Female reproductive	ALL	Human
Transcriptomic	10.1621/O24kSZBygD.1	G protein-coupled estrogen receptor	Female reproductive	ALL	House Mouse
Transcriptomic	10.1621/xbVgAAIvyF.1	G protein-coupled estrogen receptor	ALL	ALL	Human
Transcriptomic	10.1621/luVZCpT6tm.1	G protein-coupled estrogen receptor	ALL	ALL	House Mouse
Transcriptomic	10.1621/EJOOFVuj1w.1	Glucocorticoid receptor	Metabolic	Liver	House Mouse
Transcriptomic	10.1621/OASW2fSKOJ.1	Glucocorticoid receptor	Neurosensory	CNS	House Mouse
Transcriptomic	10.1621/NLcmSbOVAO.1	Glucocorticoid receptor	Respiratory	Lung	Human
Transcriptomic	10.1621/ueSYh2o5Df.1	Glucocorticoid receptor	Metabolic	Liver	Norway Rat
Transcriptomic	10.1621/U4UTCr6hpw.1	Glucocorticoid receptor	Metabolic	ALL	House Mouse
Transcriptomic	10.1621/vVANLXQU2V.1	Glucocorticoid receptor	Neurosensory	ALL	House Mouse
Transcriptomic	10.1621/mFmzqnTftL.1	Glucocorticoid receptor	Respiratory	ALL	Human
Transcriptomic	10.1621/hK9izkTNaO.1	Glucocorticoid receptor	Metabolic	ALL	Norway Rat

Transcriptomic	10.1621/XJ2jpoVxN6.1	Glucocorticoid receptor	ALL	ALL	Human
Transcriptomic	10.1621/QdbZ7Mi2ts.1	Glucocorticoid receptor	ALL	ALL	House Mouse
Transcriptomic	10.1621/nPeLunDng7.1	Glucocorticoid receptor	ALL	ALL	Norway Rat
Cistromic	10.1621/jx6Qirj3WK.1	Glucocorticoid receptor	All	All	Human
Cistromic	10.1621/tivIZ9bZss.1	Glucocorticoid receptor	All	All	House Mouse
Transcriptomic	10.1621/sxJtBux5x3.1	Insulin receptor family	ALL	ALL	Human
Cistromic	10.1621/6JgQ5eArnF.1	Jun factors	All	All	Human
Cistromic	10.1621/ZlBGp2rgVb.1	Jun factors	All	All	House Mouse
Cistromic	10.1621/ATZq8yYrNn.1	Lysine acetyltransferases (KAT)	All	All	Human
Cistromic	10.1621/BciKfFV4iR.1	Lysine acetyltransferases (KAT)	All	All	House Mouse
Transcriptomic	10.1621/gitZiqMCYO.1	Mineralocorticoid receptor	Metabolic	Liver	Norway Rat
Transcriptomic	10.1621/MhCH3hmqt6.1	Mineralocorticoid receptor	Neurosensory	CNS	House Mouse
Transcriptomic	10.1621/ZmZnYPI2e5.1	Mineralocorticoid receptor	Female reproductive	ALL	House Mouse
Transcriptomic	10.1621/5fEZrsClbG.1	Mineralocorticoid receptor	Metabolic	ALL	Norway Rat
Transcriptomic	10.1621/fwWQYgJZeZ.1	Mineralocorticoid receptor	Metabolic	ALL	House Mouse
Transcriptomic	10.1621/rJMZSL7gXX.1	Mineralocorticoid receptor	Neurosensory	ALL	House Mouse
Transcriptomic	10.1621/McszX6ox4h.1	Mineralocorticoid receptor	ALL	ALL	Human
Transcriptomic	10.1621/nHAplbKZVf.1	Mineralocorticoid receptor	ALL	ALL	House Mouse
Transcriptomic	10.1621/PSpyp4qa3f.1	Mineralocorticoid receptor	ALL	ALL	Norway Rat
Cistromic	10.1621/UOFOCLy4OH.1	Myc / Max factors	All	All	Human
Cistromic	10.1621/XOYzTIUcie.1	Myc / Max factors	All	All	House Mouse
Cistromic	10.1621/cqbwcCHQ8m.1	Myocyte enhancer factor 2	All	All	House Mouse
Cistromic	10.1621/UHrsKRsVYZ.1	Myocyte enhancer factor 2	All	All	Human
Cistromic	10.1621/EcECnqRDiC.1	Myogenic transcription factors	All	All	House Mouse
Cistromic	10.1621/xchGgbbT5W.1	Myogenic transcription factors	All	All	Human
Cistromic	10.1621/PLYeJjtqjA.1	NCoR-like	All	All	Human
Cistromic	10.1621/g3yYpnU68s.1	NCoR-like	All	All	House Mouse

Cistromic	10.1621/vFCOycZQph.1	NF-kappaB p50 subunit-like factors	All	All	Human
Cistromic	10.1621/KNKpZNMnv5.1	NF-kappaB p50 subunit-like factors	All	All	House Mouse
Transcriptomic	10.1621/wDuH53Cnco.1	Nuclear receptor coactivator (NCOA)	ALL	ALL	House Mouse
Cistromic	10.1621/Tr5qgPqJlJ.1	Nuclear receptor coactivator (NCOA)	All	All	Human
Cistromic	10.1621/ybVCQJmVml.1	Nuclear receptor coactivator (NCOA)	All	All	House Mouse
Transcriptomic	10.1621/8eSo9vz4cN.1	Peroxisome proliferator-activated receptors	Metabolic	Liver	House Mouse
Transcriptomic	10.1621/lRwZWLJbwQ.1	Peroxisome proliferator-activated receptors	Immune	Leukocytes	Human
Transcriptomic	10.1621/ETMvukJZD9.1	Peroxisome proliferator-activated receptors	Metabolic	Liver	Norway Rat
Transcriptomic	10.1621/Yi6gHbYq3N.1	Peroxisome proliferator-activated receptors	Metabolic	Adipose tissue	House Mouse
Transcriptomic	10.1621/ALwOVBZT43.1	Peroxisome proliferator-activated receptors	Metabolic	ALL	House Mouse
Transcriptomic	10.1621/jwvjWlixUi.1	Peroxisome proliferator-activated receptors	Immune	ALL	Human
Transcriptomic	10.1621/LSlhmqyj78.1	Peroxisome proliferator-activated receptors	Metabolic	ALL	Norway Rat
Transcriptomic	10.1621/Gl3VWRkh4X.1	Peroxisome proliferator-activated receptors	Metabolic	ALL	Human
Transcriptomic	10.1621/UW3ZhzPuDS.1	Peroxisome proliferator-activated receptors	Cardiovascular	ALL	House Mouse
Transcriptomic	10.1621/bzZQTlQOpH.1	Peroxisome proliferator-activated receptors	ALL	ALL	House Mouse
Transcriptomic	10.1621/ijRA3oG5pe.1	Peroxisome proliferator-activated receptors	ALL	ALL	Norway Rat
Transcriptomic	10.1621/UBtWXaQHZF.1	Peroxisome proliferator-	ALL	ALL	Human

		activated receptors			
Transcriptomic	10.1621/WW5dXhweUe.1	PPARG coactivator 1 (PPARGC1)	Metabolic	ALL	House Mouse
Transcriptomic	10.1621/yZ9E585v93.1	PPARG coactivator 1 (PPARGC1)	ALL	ALL	House Mouse
Transcriptomic	10.1621/ZOTn3KOley.1	Progesterone receptor	Female reproductive	ALL	Human
Transcriptomic	10.1621/27inpJ27Jq.1	Progesterone receptor	Female reproductive	ALL	House Mouse
Transcriptomic	10.1621/PeFrQfAMrg.1	Progesterone receptor	ALL	ALL	Human
Transcriptomic	10.1621/xBvNn58o4A.1	Progesterone receptor	ALL	ALL	House Mouse
Transcriptomic	10.1621/EDZG5FOnwY.1	Prostaglandin G/H synthases (PTGS)	Metabolic	ALL	Norway Rat
Transcriptomic	10.1621/xTwqmrHf54.1	Prostaglandin G/H synthases (PTGS)	ALL	ALL	Norway Rat
Transcriptomic	10.1621/PdMLZ24EbL.1	Retinoic acid receptors	Immune	Leukocytes	Human
Transcriptomic	10.1621/tIqJWsO24p.1	Retinoic acid receptors	Immune	ALL	Human
Transcriptomic	10.1621/Ns2ZZLrczY.1	Retinoic acid receptors	ALL	ALL	Human
Transcriptomic	10.1621/SIzL9WMaTt.1	Retinoic acid receptors	ALL	ALL	House Mouse
Transcriptomic	10.1621/LwmA2TbPyQ.1	Retinoic acid-related orphan receptors	Immune	Leukocytes	Human
Transcriptomic	10.1621/hqfGdWIPTC.1	Retinoic acid-related orphan receptors	Immune	ALL	Human
Transcriptomic	10.1621/awrRqEB2be.1	Retinoic acid-related orphan receptors	ALL	ALL	Human
Transcriptomic	10.1621/eKFA8s5YRX.1	Retinoic acid-related orphan receptors	ALL	ALL	House Mouse
Transcriptomic	10.1621/8b4MfIIdkt.1	Retinoid X receptors	ALL	ALL	Human
Cistromic	10.1621/ZCcXb1XwZx.1	SREBP factors	All	All	Human
Cistromic	10.1621/hZ3XVq6WZL.1	SREBP factors	All	All	House Mouse
Transcriptomic	10.1621/b1P9UJkmud.1	Testicular receptors (NR2C)	Immune	Leukocytes	Human
Transcriptomic	10.1621/ZHKSwOkTPu.1	Testicular receptors (NR2C)	Immune	ALL	Human
Transcriptomic	10.1621/f7u22MPXII.1	Testicular receptors (NR2C)	ALL	ALL	Human
Transcriptomic	10.1621/nAbR1IWv6J.1	Testicular receptors (NR2C)	ALL	ALL	House Mouse
Transcriptomic	10.1621/5xvSXPqcEu.1	Toll-like receptors	ALL	ALL	House Mouse

Transcriptomic	10.1621/oaotMz6MNy.1	Transient Receptor Potential channels	Metabolic	Adipose tissue	House Mouse
Transcriptomic	10.1621/WKeYxCbLdr.1	Transient Receptor Potential channels	Immune	Leukocyte s	Human
Transcriptomic	10.1621/fndsdkOvmx.1	Transient Receptor Potential channels	Metabolic	Liver	Norway Rat
Transcriptomic	10.1621/UZQpjZzvJf.1	Transient Receptor Potential channels	Female reproductive	ALL	House Mouse
Transcriptomic	10.1621/PKu3kCpb2Y.1	Transient Receptor Potential channels	Metabolic	ALL	House Mouse
Transcriptomic	10.1621/PVlTgL8YVQ.1	Transient Receptor Potential channels	Immune	ALL	Human
Transcriptomic	10.1621/XvKDi5xYoa.1	Transient Receptor Potential channels	Metabolic	ALL	Norway Rat
Transcriptomic	10.1621/Mh4VyhIH6i.1	Transient Receptor Potential channels	ALL	ALL	Human
Transcriptomic	10.1621/Y8EUiVRGkQ.1	Transient Receptor Potential channels	ALL	ALL	House Mouse
Transcriptomic	10.1621/dDFypkqQQ9.1	Transient Receptor Potential channels	ALL	ALL	Norway Rat
Transcriptomic	10.1621/hrpg2riwJX.1	Tumour necrosis factor receptors	ALL	ALL	Human
Cistromic	10.1621/3drXScdrbt.1	Two zinc-finger GATA factors	All	All	House Mouse
Cistromic	10.1621/QL7ubwiUCP.1	Two zinc-finger GATA factors	All	All	Human
Transcriptomic	10.1621/zMOR8Gqe5m.1	Vitamin D receptor	Immune	Leukocyte s	Human
Transcriptomic	10.1621/njWvJFJ96z.1	Vitamin D receptor	Immune	ALL	Human
Transcriptomic	10.1621/37wVZ5BUwH.1	Vitamin D receptor	ALL	ALL	Human
Transcriptomic	10.1621/JGzKfFMKGi.1	Xenobiotic receptors	Female reproductive	Mammary gland	Human
Transcriptomic	10.1621/JT31l2usgv.1	Xenobiotic receptors	Metabolic	Liver	Norway Rat
Transcriptomic	10.1621/hNkpcfdWNd.1	Xenobiotic receptors	Neurosensory	CNS	House Mouse
Transcriptomic	10.1621/ueW68JL3zb.1	Xenobiotic receptors	Skeletal	Bone	Human

Transcriptomic	10.1621/DXfiJcBixL.1	Xenobiotic receptors	Metabolic	Liver	House Mouse
Transcriptomic	10.1621/itszb4BKPh.1	Xenobiotic receptors	Metabolic	Liver	Human
Transcriptomic	10.1621/Hdf1zT4Nkt.1	Xenobiotic receptors	Female reproductive	Uterus	House Mouse
Transcriptomic	10.1621/rjw4P7krvE.1	Xenobiotic receptors	Female reproductive	ALL	Human
Transcriptomic	10.1621/JQPdBBBtyp.1	Xenobiotic receptors	Female reproductive	ALL	House Mouse
Transcriptomic	10.1621/oIomhnhhyY.1	Xenobiotic receptors	Metabolic	ALL	Norway Rat
Transcriptomic	10.1621/prUUGkBcCk.1	Xenobiotic receptors	Neurosensory	ALL	House Mouse
Transcriptomic	10.1621/m857iaQyJG.1	Xenobiotic receptors	Skeletal	ALL	Human
Transcriptomic	10.1621/n94e1Irpko.1	Xenobiotic receptors	Metabolic	ALL	House Mouse
Transcriptomic	10.1621/fvEP4al2wA.1	Xenobiotic receptors	Metabolic	ALL	Human
Transcriptomic	10.1621/S6lNXjjD3J.1	Xenobiotic receptors	ALL	ALL	Human
Transcriptomic	10.1621/A91BxIxhKq.1	Xenobiotic receptors	ALL	ALL	Norway Rat
Transcriptomic	10.1621/ThMZTBHQEJ.1	Xenobiotic receptors	ALL	ALL	House Mouse

Section 3. Literature and ChIP-Seq cross-validation of top-ranked transcriptomic consensome targets.

To compare consensome rankings with canonical node-target relationships, we selected the ten top ranked targets in the following consensomes: ERs-Hs-MG; the androgen receptor in human prostate gland (AR-Hs-Prostate); the glucocorticoid receptor in mouse liver (GR-Mm-Liver); and the peroxisome proliferator-activated receptor (PPAR) family in the mouse metabolic system (PPARs-Mm-Metabolic). We then searched the research literature to identify articles in which these genes had been functionally characterized as targets of these receptors. As shown, 36/40 (90%) of the most highly ranked targets across all four consensomes were validated by evidence in the research literature. Interestingly, of the six transcriptomic consensome-predicted node-target relationships for which no supporting literature evidence was found, all but one were in the 85th percentile or higher of the corresponding ChIP-Seq consensome. AR-Hs-All: Androgen receptor human all organs; ER-Hs-All: Estrogen receptors human all organs; GR-Hs-All: Glucocorticoid receptor human all organs; PPARs-Mm-Metabolic: Peroxisome proliferator activated receptors-mouse-metabolic organs; CC, percentile ranking for target in the corresponding cistromic/ChIP-Seq consensome.

Consensome	Target	Reference	CC	Consensome	Target	References	CC
**AR-Hs-All**	*ZBTB16 (PLZF)*	[1]	74	**ERs-Hs-MG**	*SGK1*	[2]	83
	UAP1	[3]	98		*TPD52L1*	-	99
	KLK3 (PSA)	[4]	99		*SIAH2*	[5]	98
	MBOAT2	-	99		*IGFBP4*	[6]	100
	C5orf30	-	61		*PRSS23*	[7]	92
	C1orf116 (SARG)	[8]	99		*HSPB8*	[9]	99
	ATAD2	[10]	100		*IL17RB*	[11]	92
	HOMER2	[12]	63		*EFNA1*	[13]	84
	TMPRSS2	[14]	90		*TFF1*	[15]	99
	PTGER4 (EP4)	[16]	93		*SLC9A3R1*	[17]	98

**GR-Mm-Liver**	*Lpin1*	18	99	**PPARs-Mm-Metabolic**	*Slc25a20*	19	93rd (Ppara), 99th (Pparg)
	Igfbp1	20	96		*Pdk4*	21	96th (Ppar), 92nd (Ppar)
	Cdkn1a (p21)	22	99		*Retsat*	23	79th (Ppara), 96th (Pparg)
	Sult1e1	24	95		*Hsdl2*	25	94th (Ppara). 97th (Pparg)
	Dusp1	26	99		*Acsl1*	27	99th (Ppara), 99th (Pparg)
	Dusp14	-	94		*Crat*	28	98th (Ppara), 97th (Pparg)
	Crybg1	-	85		*Ech1*	29	99th (Ppara), 99th (Pparg)
	Apbb3	-	98		*Acox1*	30	99th (Ppara), 92nd (Pparg)
	Atp1b1	31	94		*Decr1*	30	99th (Ppara), 99th (Pparg)
	Serpina3c	32	87		*Eci1*	33	98th (Ppara), 97th (Pparg)

REFERENCES

1 Jiang, F. & Wang, Z. Identification and characterization of PLZF as a prostatic androgen-responsive gene. *The Prostate* **59**, 426-435, doi:10.1002/pros.20000 (2004).

2 Shanmugam, I. *et al.* Serum/glucocorticoid-induced protein kinase-1 facilitates androgen receptor-dependent cell survival. *Cell death and differentiation* **14**, 2085-2094, doi:10.1038/sj.cdd.4402227 (2007).

3 Itkonen, H. M. *et al.* UAP1 is overexpressed in prostate cancer and is protective against inhibitors of N-linked glycosylation. *Oncogene* **34**, 3744-3750, doi:10.1038/onc.2014.307 (2015).

4 Kim, J. & Coetzee, G. A. Prostate specific antigen gene regulation by androgen receptor. *Journal of cellular biochemistry* **93**, 233-241, doi:10.1002/jcb.20228 (2004).

5 Frasor, J., Danes, J. M., Funk, C. C. & Katzenellenbogen, B. S. Estrogen down-regulation of the corepressor N-CoR: mechanism and implications for estrogen derepression of N-CoR-regulated genes. *Proceedings of the National Academy of Sciences of the United States of America* **102**, 13153-13157, doi:10.1073/pnas.0502782102 (2005).

6 Wang, D. Y., Fulthorpe, R., Liss, S. N. & Edwards, E. A. Identification of estrogen-responsive genes by complementary deoxyribonucleic acid microarray and characterization of a novel early estrogen-induced gene: EEIG1. *Molecular endocrinology* **18**, 402-411, doi:10.1210/me.2003-0202 (2004).

7 Chan, H. S. *et al.* Serine protease PRSS23 is upregulated by estrogen receptor alpha and associated with proliferation of breast cancer cells. *PloS one* **7**, e30397, doi:10.1371/journal.pone.0030397 (2012).

8 Steketee, K., Ziel-van der Made, A. C., van der Korput, H. A., Houtsmuller, A. B. & Trapman, J. A bioinformatics-based functional analysis shows that the specifically androgen-regulated gene SARG contains an active direct repeat androgen response element in the first intron. *Journal of molecular endocrinology* **33**, 477-491, doi:10.1677/jme.1.01478 (2004).

9 Yang, C. *et al.* Identification of cyclin D1- and estrogen-regulated genes contributing to breast carcinogenesis and progression. *Cancer research* **66**, 11649-11658, doi:10.1158/0008-5472.CAN-06-1645 (2006).

10 Duan, Z. *et al.* Developmental and androgenic regulation of chromatin regulators EZH2 and ANCCA/ATAD2 in the prostate Via MLL histone methylase complex. *The Prostate* **73**, 455-466, doi:10.1002/pros.22587 (2013).

11 Wang, Z. *et al.* The prognostic biomarkers HOXB13, IL17BR, and CHDH are regulated by estrogen in breast cancer. *Clinical cancer research : an official journal of the American Association for Cancer Research* **13**, 6327-6334, doi:10.1158/1078-0432.CCR-07-0310 (2007).

12 Kurmis, A. A., Yang, F., Welch, T. R., Nickols, N. G. & Dervan, P. B. A Pyrrole-Imidazole Polyamide Is Active against Enzalutamide-Resistant Prostate Cancer. *Cancer research* **77**, 2207-2212, doi:10.1158/0008-5472.CAN-16-2503 (2017).

13 Andruska, N. D. *et al.* Estrogen receptor alpha inhibitor activates the unfolded protein response, blocks protein synthesis, and induces tumor regression. *Proceedings of the National Academy of Sciences of the United States of America* **112**, 4737-4742, doi:10.1073/pnas.1403685112 (2015).

14 Lucas, J. M. *et al.* The androgen-regulated protease TMPRSS2 activates a proteolytic cascade involving components of the tumor microenvironment and promotes prostate cancer metastasis. *Cancer Discov* **4**, 1310-1325, doi:10.1158/2159-8290.CD-13-1010 (2014).

15 Sun, J. M. *et al.* Estrogen regulation of trefoil factor 1 expression by estrogen receptor alpha and Sp proteins. *Experimental cell research* **302**, 96-107, doi:10.1016/j.yexcr.2004.08.015 (2005).

16 Terada, N. *et al.* Identification of EP4 as a potential target for the treatment of castration-resistant prostate cancer using a novel xenograft model. *Cancer research* **70**, 1606-1615, doi:10.1158/0008-5472.CAN-09-2984 (2010).

17 Yang, L. *et al.* Na(+)/H(+) exchanger regulatory factor 1 (NHERF1) is required for the estradiol-dependent increase of phosphatase and tensin homolog (PTEN) protein expression. *Endocrinology* **152**, 4537-4549, doi:10.1210/en.2011-1207 (2011).

18 Zhang, P., O'Loughlin, L., Brindley, D. N. & Reue, K. Regulation of lipin-1 gene expression by glucocorticoids during adipogenesis. *Journal of lipid research* **49**, 1519-1528, doi:10.1194/jlr.M800061-JLR200 (2008).

19 Tachibana, K. *et al.* Regulation of the human SLC25A20 expression by peroxisome proliferator-activated receptor alpha in human hepatoblastoma cells. *Biochemical and biophysical research communications* **389**, 501-505, doi:10.1016/j.bbrc.2009.09.018 (2009).

20 Goswami, R., Lacson, R., Yang, E., Sam, R. & Unterman, T. Functional analysis of glucocorticoid and insulin response sequences in the rat insulin-like growth factor-binding protein-1 promoter. *Endocrinology* **134**, 736-743, doi:10.1210/endo.134.2.7507835 (1994).
21 Wu, P., Peters, J. M. & Harris, R. A. Adaptive increase in pyruvate dehydrogenase kinase 4 during starvation is mediated by peroxisome proliferator-activated receptor alpha. *Biochemical and biophysical research communications* **287**, 391-396, doi:10.1006/bbrc.2001.5608 (2001).
22 Cha, H. H. *et al.* Glucocorticoids stimulate p21 gene expression by targeting multiple transcriptional elements within a steroid responsive region of the p21waf1/cip1 promoter in rat hepatoma cells. *The Journal of biological chemistry* **273**, 1998-2007 (1998).
23 Schupp, M. *et al.* Retinol saturase promotes adipogenesis and is downregulated in obesity. *Proceedings of the National Academy of Sciences of the United States of America* **106**, 1105-1110, doi:10.1073/pnas.0812065106 (2009).
24 Gong, H. *et al.* Glucocorticoids antagonize estrogens by glucocorticoid receptor-mediated activation of estrogen sulfotransferase. *Cancer research* **68**, 7386-7393, doi:10.1158/0008-5472.CAN-08-1545 (2008).
25 Adhikary, T. *et al.* Genomewide analyses define different modes of transcriptional regulation by peroxisome proliferator-activated receptor-beta/delta (PPARbeta/delta). *PloS one* **6**, e16344, doi:10.1371/journal.pone.0016344 (2011).
26 Shipp, L. E. *et al.* Transcriptional regulation of human dual specificity protein phosphatase 1 (DUSP1) gene by glucocorticoids. *PloS one* **5**, e13754, doi:10.1371/journal.pone.0013754 (2010).
27 Martin, G., Schoonjans, K., Lefebvre, A. M., Staels, B. & Auwerx, J. Coordinate regulation of the expression of the fatty acid transport protein and acyl-CoA synthetase genes by PPARalpha and PPARgamma activators. *The Journal of biological chemistry* **272**, 28210-28217 (1997).
28 Kienesberger, K., Pordes, A. G., Volk, T. G. & Hofbauer, R. L-carnitine and PPARalpha-agonist fenofibrate are involved in the regulation of Carnitine Acetyltransferase (CrAT) mRNA levels in murine liver cells. *BMC genomics* **15**, 514, doi:10.1186/1471-2164-15-514 (2014).
29 Reddy, J. K. *et al.* Transcription regulation of peroxisomal fatty acyl-CoA oxidase and enoyl-CoA hydratase/3-hydroxyacyl-CoA dehydrogenase in rat liver by peroxisome proliferators. *Proceedings of the National Academy of Sciences of the United States of America* **83**, 1747-1751 (1986).
30 Rakhshandehroo, M., Hooiveld, G., Muller, M. & Kersten, S. Comparative analysis of gene regulation by the transcription factor PPARalpha between mouse and human. *PloS one* **4**, e6796, doi:10.1371/journal.pone.0006796 (2009).
31 Huynh, T. P. *et al.* Glucocorticoids suppress renal cell carcinoma progression by enhancing Na,K-ATPase beta-1 subunit expression. *PloS one* **10**, e0122442, doi:10.1371/journal.pone.0122442 (2015).
32 Asada, M. *et al.* DNA binding-dependent glucocorticoid receptor activity promotes adipogenesis via Kruppel-like factor 15 gene expression. *Lab Invest* **91**, 203-215, doi:10.1038/labinvest.2010.170 (2011).
33 Rakhshandehroo, M., Knoch, B., Muller, M. & Kersten, S. Peroxisome proliferator-activated receptor alpha target genes. *PPAR research* **2010**, doi:10.1155/2010/612089 (2010).

Section 4. Gene targets in the 99th percentile of the mouse All nodes liver transcriptomic consensome.

This list represents the top 1% of genes that are significantly differentially expressed in expression profiling experiments in a murine hepatic biosample, irrespective of the perturbed signaling node. Six digit numbers refer to OMIM record entries and can be resolved at the URL of the format https://omim.org/entry/123456

Gene name	Mm Approved Symbol	Percentile	CPV	Metabolic enzyme	Metabolic disease associated with deficiency in human ortholog	Pathway in which enzyme regulates rate-limiting step
ectonucleoside triphosphate diphosphohydrolase 5	*Entpd5*	99.99	9.81E-114	Yes		
acyl-CoA thioesterase 1	*Acot1*	99.99	1.59E-111	Yes		
Epoxide hydroxylase 1, microsomal xenobiotic	*Ephx1*	99.98	7.27E-107	Yes	Hypercholanemia, familial, 607748 , Autosomal recessive	
Aldehyde dehydrogenase 3 family, member A2 (fatty aldehyde dehydrogenase)	*Aldh3a2*	99.98	2.11E-103	Yes	Sjogren-Larsson syndrome, 270200 , Autosomal recessive	Oxidation of fatty aldehydes to fatty acids
retinol saturase (all trans retinol 13,14 reductase)	*Retsat*	99.98	1.85E-99	Yes		
aldehyde oxidase 3	*Aox3*	99.97	1.702E-94	Yes		
Proline dehydrogenase (proline oxidase)	*Prodh*	99.97	2.547E-94	Yes	Hyperprolinemia, type I, 239500 , Autosomal recessive; {Schizophrenia, susceptibility to, 4}, 600850 , Autosomal dominant	Oxidation of proline to glutamate
enoyl coenzyme A hydratase 1, peroxisomal	*Ech1*	99.96	5.121E-91	Yes		
glutathione S-transferase, mu 4	*Gstm4*	99.96	1.373E-90	Yes		
vanin 1	*Vnn1*	99.96	4.18E-89			
Enoyl-Coenzyme A, hydratase/3-hydroxyacyl Coenzyme A dehydrogenase	*Ehhadh*	99.95	4.703E-89	Yes	Fanconi renotubular syndrome 3, 615605 , Autosomal dominant	
ATP-binding cassette, sub-family C (CFTR/MRP), member 3	*Abcc3*	99.95	1.39E-88			

malic enzyme 1, NADP(+)-dependent, cytosolic	*Me1*	99.94	2.2E-88	Yes		
dexamethasone-induced transcript	*Dexi*	99.94	2.2E-88			
MAPK regulated corepressor interacting protein 2	*Mcrip2*	99.94	2.2E-88			
peroxisomal biogenesis factor 11 alpha	*Pex11a*	99.93	4.272E-88			
cytochrome P450, family 2, subfamily b, polypeptide 10	*Cyp2b10*	99.93	1.247E-87			
acetyl-Coenzyme A acyltransferase 1B	*Acaa1b*	99.93	6.09E-87	Yes		
Cystathionine beta-synthase	*Cbs*	99.92	6.478E-87	Yes	Homocystinuria, B6-responsive and nonresponsive types, 236200 , Autosomal recessive; Thrombosis, hyperhomocysteinemic, 236200 , Autosomal recessive	Trans-sulfuration pathway
pyruvate dehydrogenase kinase 4	*Pdk4*	99.92	1.111E-86	Yes		Regulates PDH, which catalyzes the rate limiting oxidation of pyruvate to AcCoA
perilipin 5	*Plin5*	99.91	1.179E-86			
Histidine ammonia-lyase (histidase)	*Hal*	99.91	3.56E-86	Yes	[Histidinemia], 235800 , Autosomal recessive, Autosomal dominant	
glutathione S-transferase, alpha 2 (Yc2)	*Gsta2*	99.91	8.583E-86	Yes		
glutathione S-transferase, theta 2	*Gstt2*	99.9	1.066E-85	Yes		
serum amyloid A 4	*Saa4*	99.9	1.066E-85			
chymotrypsin-like elastase family, member 1	*Cela1*	99.89	1.577E-84			
Argininosuccinate lyase	*Asl*	99.89	1.543E-83	Yes	Argininosuccinic aciduria, 207900 , Autosomal recessive	Arginine synthesis
nicotinamide N-methyltransferase	*Nnmt*	99.89	3.633E-83			
aldehyde oxidase 1	*Aox1*	99.88	7.682E-83	Yes		
P450 (cytochrome) oxidoreductase	*Por*	99.88	1.013E-82			
solute carrier family 25 (mitochondrial	*Slc25a20*	99.88	1.225E-81			

carnitine/acylcarnitine translocase), member 20						
carbonyl reductase 1	*Cbr1*	99.87	3.317E-81	Yes		Conversion of PGE2 to PGF2alpha
3-hydroxy-3-methylglutaryl-Coenzyme A synthase 2, mitochondrial	*Hmgcs2*	99.87	1.233E-80	Yes	HMG-CoA synthase-2 deficiency, 605911 , Autosomal recessive	Ketogenesis
metallothionein 2	*Mt2*	99.87	1.233E-80			
G0/G1 switch gene 2	*G0s2*	99.86	2.032E-80			
choline dehydrogenase	*Chdh*	99.86	3.335E-80	Yes		
aquaporin 8	*Aqp8*	99.85	9.659E-80			
CD36 molecule	*Cd36*	99.85	9.659E-80			
Pyruvate kinase, liver and RBC type	*Pklr*	99.84	1.048E-79	Yes	Adenosine triphosphate, elevated, of erythrocytes, 102900 , Autosomal dominant; Pyruvate kinase deficiency, 266200 , Autosomal recessive	Glycolysis
growth differentiation factor 15	*Gdf15*	99.84	1.048E-79			
hydroxysteroid dehydrogenase like 2	*Hsdl2*	99.84	1.05E-79	Yes		
glycerol-3-phosphate acyltransferase 3	*Gpat3*	99.83	2.599E-79			
serine (or cysteine) peptidase inhibitor, clade A (alpha-1 antiproteinase, antitrypsin), member 7	*Serpina7*	99.83	2.599E-79			
glutathione S-transferase, mu 2	*Gstm2*	99.83	4.228E-79	Yes		
transmembrane protein 98	*Tmem98*	99.82	8.52E-79			
solute carrier organic anion transporter family, member 1a4	*Slco1a4*	99.82	1.438E-78			
arginine vasopressin receptor 1A	*Avpr1a*	99.81	1.451E-78			
Epoxide hydrolase 2, cytoplasmic	*Ephx2*	99.81	6.069E-78	Yes	{Hypercholesterolemia, familial, due to LDLR defect, modifier of}, 143890 , Autosomal dominant	
cytochrome P450, family 1, subfamily a, polypeptide 2	*Cyp1a2*	99.81	1.006E-77			
glutathione S-transferase, mu 3	*Gstm3*	99.8	1.136E-77	Yes		

cysteine sulfinic acid decarboxylase	*Csad*	99.8	3.214E-77	Yes		Taurine biosynthesis
coagulation factor VII	*F7*	99.79	3.529E-77			
enoyl-Coenzyme A delta isomerase 2	*Eci2*	99.79	8.586E-77	Yes		
aldo-keto reductase family 1, member C19	*Akr1c19*	99.79	8.74E-77			
glycosylphosphatidylinositol specific phospholipase D1	*Gpld1*	99.78	2.323E-76			
aldehyde dehydrogenase 1 family member A7	*Aldh1a7*	99.78	2.687E-76	Yes		Retinoic acid biosynthesis
Glutamic-oxaloacetic transaminase-1, soluble (EC 2.6.1.1)	*Got1*	99.78	4.301E-76	Yes	Aspartate aminotransferase, serum level of, QTL1, 614419	
ST3 beta-galactoside alpha-2,3-sialyltransferase 5	*St3gal5*	99.77	6.221E-76			
cell death-inducing DFFA-like effector c	*Cidec*	99.77	1.197E-75			
enoyl-Coenzyme A delta isomerase 1	*Eci1*	99.76	1.959E-75	Yes		
ubiquitin specific peptidase 18	*Usp18*	99.76	1.959E-75			
UDP-glucose dehydrogenase	*Ugdh*	99.76	2.622E-75	Yes		
peroxisomal membrane protein 4	*Pxmp4*	99.75	4.341E-75			
acyl-CoA thioesterase 4	*Acot4*	99.75	7.167E-75	Yes		
solute carrier family 22 (organic cation transporter), member 5	*Slc22a5*	99.74	1.644E-74			
lysophosphatidylcholine acyltransferase 3	*Lpcat3*	99.74	8.45E-74	Yes		
chemokine (C-C motif) ligand 9	*Ccl9*	99.73	2.226E-73			
protein S (alpha)	*Pros1*	99.73	2.226E-73			
glutathione S-transferase, alpha 4	*Gsta4*	99.73	2.358E-73	Yes		
cellular repressor of E1A-stimulated genes 1	*Creg1*	99.72	3.138E-73			
cAMP responsive element binding protein 3-like 3	*Creb3l3*	99.72	5.143E-73			
neuritin 1	*Nrn1*	99.72	8.433E-73			
early growth response 1	*Egr1*	99.71	9.47E-73			

carbonic anhydrase 14	*Car14*	99.71	1.843E-72			
protein phosphatase 1, regulatory subunit 3C	*Ppp1r3c*	99.71	2.4E-72			
transmembrane protein 97	*Tmem97*	99.7	2.969E-72			
androgen dependent TFPI regulating protein	*Adtrp*	99.69	3.09E-72			
PERP, TP53 apoptosis effector	*Perp*	99.69	4.324E-72			
inhibin beta-C	*Inhbc*	99.69	4.772E-72			
solute carrier family 46, member 3	*Slc46a3*	99.68	7.042E-72			
acyl-CoA synthetase long-chain family member 1	*Acsl1*	99.68	7.651E-72	Yes		
RIKEN cDNA 4931406C07 gene	*4931406C07Rik*	99.67	3.903E-71			
transcription elongation factor A (SII), 3	*Tcea3*	99.67	3.903E-71			
phospholysine phosphohistidine inorganic pyrophosphate phosphatase	*Lhpp*	99.67	5.868E-71	Yes		
2,4-dienoyl-CoA reductase 1	*Decr1*	99.66	6.234E-71	Yes	2,4-dienoyl-CoA reductase deficiency (DECRD)	Unsaturated fatty acid oxidation in mitochondria
solute carrier family 2 (facilitated glucose transporter), member 2	*Slc2a2*	99.66	6.234E-71			
cytochrome P450, family 2. subfamily c, polypeptide 37	*Cyp2c37*	99.66	6.382E-71			
carboxypeptidase N, polypeptide 2	*Cpn2*	99.65	9.496E-71			
phospholipase A2 group XIIA	*Pla2g12a*	99.65	3.175E-70	Yes		Linoleic acid metabolism
hairy and enhancer of split 6	*Hes6*	99.64	5.054E-70			
pipecolic acid oxidase	*Pipox*	99.64	5.054E-70			
perilipin 2	*Plin2*	99.64	8.025E-70			
ATP-binding cassette, sub-family A (ABC1), member 8a	*Abca8a*	99.63	1.261E-69			
ATP binding cassette subfamily G member 5	*Abcg5*	99.63	1.261E-69			
cytochrome P450, family 2, subfamily c, polypeptide 54	*Cyp2c54*	99.62	1.529E-69			

GrpE-like 2, mitochondrial	*Grpel2*	99.62	1.616E-69			
carboxylesterase 1E	*Ces1e*	99.62	2.023E-69	Yes		
fat storage-inducing transmembrane protein 2	*Fitm2*	99.61	4.981E-69			
coagulation factor XI	*F11*	99.61	5.164E-69			
solute carrier family 7 (cationic amino acid transporter, y+ system), member 2	*Slc7a2*	99.61	6.448E-69			
cytochrome P450, family 4, subfamily f, polypeptide 14	*Cyp4f14*	99.6	7.904E-69			
lectin, galactoside-binding, soluble, 3 binding protein	*Lgals3bp*	99.6	9.229E-69			
Hydroxy-delta-5-steroid dehydrogenase, 3 beta- and steroid delta-isomerase, type 2 (adrenal, gonadal)	*Hsd3b2*	99.59	1.018E-68	Yes	Adrenal hyperplasia, congenital, due to 3-beta-hydroxysteroid dehydrogenase 2 deficiency, 201810 , Autosomal recessive	Aldosterone production
glutathione S-transferase pi 3	*Gstp3*	99.59	1.032E-68			
kallikrein B, plasma 1	*Klkb1*	99.59	1.275E-68			
glucosamine-6-phosphate deaminase 1	*Gnpda1*	99.58	1.649E-68	Yes		Hexosamine biosynthesis
cytochrome P450, family 4, subfamily a, polypeptide 31	*Cyp4a31*	99.58	4.048E-68			
amidohydrolase domain containing 1	*Amdhd1*	99.57	5.809E-68	Yes		
protease, serine 8 (prostasin)	*Prss8*	99.57	5.809E-68			
retinol dehydrogenase 16	*Rdh16*	99.56	8.105E-68	Yes		All-trans retinoic acid biosynthesis
tubulin folding cofactor E-like	*Tbcel*	99.56	8.105E-68			
serum amyloid P-component	*Apcs*	99.56	1.272E-67			
abhydrolase domain containing 6	*Abhd6*	99.56	1.338E-67	Yes		2-arachidonoglycerol biosynthesis
out at first homolog	*Oaf*	99.55	1.679E-67			
flavin containing monooxygenase 5	*Fmo5*	99.55	1.722E-67			
Sulfite oxidase	*Suox*	99.54	2.124E-67	Yes	Sulfite oxidase deficiency, 272300 , Autosomal recessive	
oxidative stress induced growth inhibitor 1	*Osgin1*	99.54	3.11E-67			

progestin and adipoQ receptor family member IX	*Paqr9*	99.54	3.114E-67			
Sarcosine dehydrogenase	*Sardh*	99.53	6.419E-67	Yes	[Sarcosinemia], 268900 , Autosomal recessive	
solute carrier family 41, member 2	*Slc41a2*	99.53	8.384E-67			
UDP glucuronosyltransferase 2 family, polypeptide B35	*Ugt2b35*	99.52	9.25E-67			
cytochrome P450, family 4, subfamily a, polypeptide 14	*Cyp4a14*	99.52	1.004E-66			
peptidoglycan recognition protein 2	*Pglyrp2*	99.52	1.29E-66			
acyl-CoA synthetase long-chain family member 5	*Acsl5*	99.51	1.318E-66	Yes		
UDP-glucose pyrophosphorylase 2	*Ugp2*	99.51	1.566E-66	Yes		
homocysteine-inducible, endoplasmic reticulum stress-inducible, ubiquitin-like domain member 1	*Herpud1*	99.51	2.039E-66			
abhydrolase domain containing 14b	*Abhd14b*	99.5	2.694E-66	Yes		
biliverdin reductase B (flavin reductase (NADPH))	*Blvrb*	99.5	2.694E-66	Yes		
RIKEN cDNA 2010003K11 gene	*2010003K11Rik*	99.49	4.366E-66			
carboxylesterase 1G	*Ces1g*	99.49	5.056E-66	Yes		
cytochrome P450, family 39, subfamily a, polypeptide 1	*Cyp39a1*	99.49	6.663E-66			
cytochrome P450, family 4, subfamily a, polypeptide 12a	*Cyp4a12a*	99.48	6.962E-66			
tubulin, beta 4B class IVB	*Tubb4b*	99.48	9.062E-66			
Dimethylglycine dehydrogenase	*Dmgdh*	99.46	1.224E-65	Yes	Dimethylglycine dehydrogenase deficiency, 605850 , Autosomal recessive	Betaine metabolism
monoglyceride lipase	*Mgll*	99.46	1.224E-65	Yes		Monoacylglycerol catabolism
Guanidinoacetate methyltransferase	*Gamt*	99.46	1.224E-65	Yes	Cerebral creatine deficiency syndrome 2, 612736 , Autosomal recessive	
Lipin 2	*Lpin2*	99.46	1.224E-65	Yes	Majeed syndrome, 609628	

RAB9, member RAS oncogene family	*Rab9*	99.46	1.224E-65			
sulfiredoxin 1 homolog (S. cerevisiae)	*Srxn1*	99.46	1.224E-65			
nuclear factor, interleukin 3, regulated	*Nfil3*	99.45	2.938E-65			
Catechol-O-methyltransferase	*Comt*	99.44	5.266E-65	Yes	{Panic disorder, susceptibility to}, 167870 , Autosomal dominant; {Schizophrenia, susceptibility to}, 181500 , Autosomal dominant	
cytochrome P450, family 2, subfamily c, polypeptide 70	*Cyp2c70*	99.44	5.266E-65			
phosphatidylinositol glycan anchor biosynthesis, class P	*Pigp*	99.44	5.266E-65			
RDH16 family member 2	*Rdh16f2*	99.44	7.106E-65			
pleckstrin homology domain containing, family F (with FYVE domain) member 1	*Plekhf1*	99.43	8.915E-65			
Acyl-Coenzyme A dehydrogenase, C-4 to C-12 straight chain	*Acadm*	99.43	9.52E-65	Yes	Acyl-CoA dehydrogenase, medium chain, deficiency of, 201450 , Autosomal recessive	Medium-chain fatty acid β-oxidation
angiotensinogen (serpin peptidase inhibitor, clade A, member 8)	*Agt*	99.42	1.471E-64			
hepatocyte growth factor activator	*Hgfac*	99.42	1.471E-64			
orosomucoid 2	*Orm2*	99.41	2.268E-64			
retinol binding protein 1, cellular	*Rbp1*	99.41	2.268E-64			
transmembrane protein 37	*Tmem37*	99.41	2.654E-64			
ribonuclease, RNase A family 4	*Rnase4*	99.4	3.491E-64			
2,4-dienoyl-CoA reductase 2	*Decr2*	99.4	4.138E-64	Yes		Unsaturated fatty acid oxidation in mitochondria
angiogenin, ribonuclease, RNase A family, 5	*Ang*	99.4	4.138E-64			
ATP-binding cassette, sub-family C (CFTR/MRP), member 4	*Abcc4*	99.39	4.912E-64			
complement component 8, alpha polypeptide	*C8a*	99.39	6.436E-64			

solute carrier family 37 (glucose-6-phosphate transporter), member 4	*Slc37a4*	99.39	7.361E-64			
serine dehydratase	*Sds*	99.38	8.204E-64	Yes		
thymidine kinase 1	*Tk1*	99.38	1.741E-63	Yes		Nucleotide salvage pathway
fatty acid binding protein 2, intestinal	*Fabp2*	99.37	1.764E-63			
Galactokinase-1	*Galk1*	99.37	2.669E-63	Yes	Galactokinase deficiency with cataracts, 230200 , Autosomal recessive	
indolethylamine N-methyltransferase	*Inmt*	99.37	2.669E-63			
MACRO domain containing 1	*Macrod1*	99.36	3.233E-63	Yes		
BAI1-associated protein 2-like 1	*Baiap2l1*	99.36	3.233E-63			
fibroblast growth factor 21	*Fgf21*	99.35	4.083E-63			
gulonolactone (L-) oxidase	*Gulo*	99.35	5.01E-63	Yes		Ascorbic acid biosynthesis
glutathione S-transferase, theta 3	*Gstt3*	99.35	6.231E-63	Yes		
monocyte to macrophage differentiation-associated	*Mmd*	99.34	7.746E-63			
Aminolevulinate, delta-, synthase-2	*Alas2*	99.34	8.682E-63	Yes	Anemia, sideroblastic, 1, 300751 , X-linked recessive; Protoporphyria, erythropoietic, X-linked, 300752 , X-linked	Heme biosynthesis
carboxylesterase 1D	*Ces1d*	99.33	1.329E-62	Yes		
myosin IB	*Myo1b*	99.33	1.329E-62			
growth arrest and DNA-damage-inducible 45 beta	*Gadd45b*	99.32	1.394E-62			
low density lipoprotein receptor-related protein 4	*Lrp4*	99.32	1.394E-62			
NAD(P)H dehydrogenase, quinone 1	*Nqo1*	99.32	1.394E-62			
Lipoprotein lipase	*Lpl*	99.32	1.442E-62	Yes	Combined hyperlipidemia, familial, 144250 , Autosomal dominant; [High density lipoprotein cholesterol level QTL 11] ; Lipoprotein lipase deficiency, 238600 , Autosomal recessive	Hydrolysis of core TGs from TG-rich lipoproteins

macrophage expressed gene 1	*Mpeg1*	99.31	1.449E-62			
ChaC, cation transport regulator 1	*Chac1*	99.31	1.839E-62			
aldehyde dehydrogenase 1 family member A1	*Aldh1a1*	99.29	2.03E-62	Yes		Retinoic acid biosynthesis
glutathione S-transferase, mu 1	*Gstm1*	99.29	2.03E-62	Yes		
interferon induced transmembrane protein 3	*Ifitm3*	99.29	2.03E-62			
phosphatidylcholine transfer protein	*Pctp*	99.29	2.03E-62			
solute carrier family 29 (nucleoside transporters), member 1	*Slc29a1*	99.29	2.03E-62			
solute carrier family 6 (neurotransmitter transporter, betaine/GABA), member 12	*Slc6a12*	99.29	2.03E-62			
hydroxysteroid (17-beta) dehydrogenase 2	*Hsd17b2*	99.28	2.303E-62	Yes		
Alpha-aminoadipic semialdehyde synthase	*Aass*	99.28	3.094E-62	Yes	Hyperlysinemia, 238700 , Autosomal recessive; Saccharopinuria, 268700 (1), Autosomal recessive	Lysine catabolism
Phenylalanine hydroxylase	*Pah*	99.28	3.094E-62	Yes	[Hyperphenylalaninemia, non-PKU mild], 261600 , Autosomal recessive; Phenylketonuria, 261600 , Autosomal recessive	Phenylalanine catabolism
RAB30, member RAS oncogene family	*Rab30*	99.27	3.877E-62			
zinc binding alcohol dehydrogenase, domain containing 2	*Zadh2*	99.26	5.972E-62	Yes		
phenazine biosynthesis-like protein domain containing 2	*Pbld2*	99.26	5.972E-62			
cilia and flagella associated protein 20	*Cfap20*	99.26	6.611E-62			
PDZ domain containing 1	*Pdzk1*	99.26	6.611E-62			
Tyrosine aminotransferase, soluble	*Tat*	99.25	7.14E-62	Yes	Tyrosinemia, type II, 276600 , Autosomal recessive	Tyrosine catabolism
lymphocyte antigen 6 complex, locus D	*Ly6d*	99.25	9.178E-62			
transglutaminase 1, K polypeptide	*Tgm1*	99.24	1.081E-61	Yes	Autosomal recessive congenital ichthyosis	Formation of cross-linked protein envelope in

						terminal differentiation of skin
RIKEN cDNA 4931408D14 gene	*4931408D14Rik*	99.24	1.669E-61			
keratin 23	*Krt23*	99.24	1.701E-61			
Xanthine dehydrogenase (xanthine oxidase)	*Xdh*	99.23	2.331E-61	Yes	Xanthinuria, type I, 278300 , Autosomal recessive	Purine metabolism
cysteine rich protein 2	*Crip2*	99.23	2.331E-61			
glycerophosphodiester phosphodiesterase 1	*Gde1*	99.23	2.331E-61			
RIKEN cDNA B230114P17 gene	*B230114P17Rik*	99.22	2.747E-61			
retinol dehydrogenase 9	*Rdh9*	99.22	2.983E-61	Yes		All-trans retinoic acid biosynthesis
Acyl-Coenzyme A oxidase 1, palmitoyl	*Acox1*	99.21	3.532E-61	Yes	Peroxisomal acyl-CoA oxidase deficiency, 264470 , Autosomal recessive	Peroxisomal fatty acid β-oxidation pathway
retinoic acid receptor responder (tazarotene induced) 1	*Rarres1*	99.21	3.844E-61			
glutathione peroxidase 7	*Gpx7*	99.2	4.674E-61	Yes		
insulin-like growth factor binding protein 2	*Igfbp2*	99.2	5.341E-61			
metallothionein 1	*Mt1*	99.2	5.685E-61			
carboxylesterase 1F	*Ces1f*	99.19	7.009E-61	Yes		
5-oxoprolinase (ATP-hydrolysing)	*Oplah*	99.19	7.009E-61			
cyclin-dependent kinase inhibitor 1A (P21)	*Cdkn1a*	99.18	1.214E-60			
lectin, galactose binding, soluble 4	*Lgals4*	99.18	1.323E-60			
cytidine deaminase	*Cda*	99.18	1.632E-60	Yes		
synaptosomal-associated protein, 47	*Snap47*	99.17	1.745E-60			
Alkaline phosphatase, liver/bone/kidney	*Alpl*	99.17	1.865E-60	Yes	Hypophosphatasia, adult, 146300 , Autosomal recessive, Autosomal dominant; Hypophosphatasia, childhood, 241510 , Autosomal recessive; Hypophosphatasia, infantile, 241500 , Autosomal recessive; Odontohypophosphatasia,	

					146300 , Autosomal recessive, Autosomal dominant	
ATP binding cassette subfamily G member 2 (Junior blood group)	*Abcg2*	99.17	2.043E-60			
high mobility group box 3	*Hmgb3*	99.16	3.488E-60			
dehydrogenase/reductase (SDR family) member 4	*Dhrs4*	99.15	3.971E-60	Yes		
cytochrome P450, family 3, subfamily a, polypeptide 13	*Cyp3a13*	99.15	3.971E-60			
phospholipid scramblase 2	*Plscr2*	99.15	3.971E-60			
carboxylesterase 2G	*Ces2g*	99.15	5.703E-60	Yes		
complement component 2 (within H-2S)	*C2*	99.14	5.972E-60			
carboxylesterase 2E	*Ces2e*	99.14	8.097E-60	Yes		
major facilitator superfamily domain containing 2A	*Mfsd2a*	99.13	8.933E-60			
inhibin beta-E	*Inhbe*	99.13	9.084E-60			
A kinase (PRKA) interacting protein 1	*Akip1*	99.13	1.734E-59			
leukocyte cell-derived chemotaxin 2	*Lect2*	99.12	2.817E-59			
Heme oxygenase 1	*Hmox1*	99.11	2.935E-59	Yes	Heme oxygenase-1 deficiency, 614034 ; {Pulmonary disease, chronic obstructive, susceptibility to}, 606963	Heme degradation
Aconitase, mitochondrial	*Aco2*	99.11	2.935E-59	Yes	Infantile cerebellar-retinal degeneration, 614559 , Autosomal recessive; ?Optic atrophy 9, 616289 , Autosomal recessive	
solute carrier family 9 (sodium/hydrogen exchanger), member 3 regulator 1	*Slc9a3r1*	99.11	2.935E-59			
SPARC related modular calcium binding 1	*Smoc1*	99.11	2.935E-59			
translocase of inner mitochondrial membrane 8A1	*Timm8a1*	99.11	2.935E-59			

doublecortin-like kinase 3	*Dclk3*	99.1	4.031E-59			
Retinol dehydrogenase-5	*Rdh5*	99.09	4.398E-59	Yes	Fundus albipunctatus, 136880 , Autosomal recessive, Autosomal dominant	All-trans retinoic acid synthesis
etoposide induced 2.4 mRNA	*Ei24*	99.09	4.398E-59			
interferon regulatory factor 7	*Irf7*	99.09	4.398E-59			
cytidine 5'-triphosphate synthase	*Ctps*	99.08	7.853E-59			
Cystathionine gamma-lyase	*Cth*	99.07	9.208E-59	Yes	Cystathioninuria, 219500 , Autosomal recessive; Homocysteine, total plasma, elevated	Cysteine synthesis
N-acetyltransferase 8 (GCN5-related)	*Nat8*	99.07	9.208E-59			
plasma membrane proteolipid	*Pllp*	99.07	9.208E-59			
serine (or cysteine) peptidase inhibitor, clade F, member 2	*Serpinf2*	99.07	9.208E-59			
solute carrier family 17 (sodium phosphate), member 2	*Slc17a2*	99.07	9.208E-59			
B cell translocation gene 2, anti-proliferative	*Btg2*	99.06	1.39E-58			
Malonyl-CoA decarboxylase	*Mlycd*	99.06	1.441E-58	Yes	Malonyl-CoA decarboxylase deficiency, 248360 , Autosomal recessive	Conversion of malonyl-CoA to acetyl-CoA and CO2
proteoglycan 4 (megakaryocyte stimulating factor, articular superficial zone protein)	*Prg4*	99.05	1.841E-58	Yes		
basic helix-loop-helix domain containing, class B9	*Bhlhb9*	99.05	1.981E-58			
aldehyde dehydrogenase 16 family, member A1	*Aldh16a1*	99.04	2.808E-58			
immediate early response 2	*Ier2*	99.04	2.808E-58			
methyltransferase like 7B	*Mettl7b*	99.04	2.808E-58			
Carbamoyl-phosphate synthetase 1, mitochondrial	*Cps1*	99.03	3.148E-58	Yes	Carbamoylphosphate synthetase I deficiency, 237300 , Autosomal recessive; {Pulmonary hypertension, neonatal, susceptibility to}, 615371 ; {Venoocclusive disease after bone marrow transplantation}	Urea cycle

Ornithine aminotransferase	*Oat*	99.03	3.148E-58	Yes	Gyrate atrophy of choroid and retina with or without ornithinemia, 258870 , Autosomal recessive	
Ketohexokinase (fructokinase)	*Khk*	99.02	3.219E-58	Yes	[Fructosuria], 229800 , Autosomal recessive	Conversion of fructose to fructose-1-phosphate
Carnitine palmitoyltransferase II	*Cpt2*	99.02	3.219E-58	Yes	CPT II deficiency, infantile, 600649 , Autosomal recessive; CPT II deficiency, lethal neonatal, 608836 , Autosomal recessive; CPT II deficiency, myopathic, stress-induced, 255110 , Autosomal recessive, Autosomal dominant; {Encephalopathy, acute, infection-induced, 4, susceptibility to}, 614212 , Autosomal recessive, Autosomal dominant	Mitochondrial fatty acid beta-oxidation
Isocitrate dehydrogenase, soluble	*Idh1*	99.02	3.219E-58	Yes	{Glioma, susceptibility to, somatic}, 137800	TCA cycle
17-beta-hydroxysteroid dehydrogenase X	*Hsd17b10*	99.02	3.219E-58	Yes	HSD10 mitochondrial disease, 300438 , X-linked dominant	
carboxymethylenebutenolidase-like (Pseudomonas)	*Cmbl*	99.02	3.219E-58			
collagen, type XVIII, alpha 1	*Col18a1*	99.02	3.219E-58			
poly (ADP-ribose) polymerase family, member 16	*Parp16*	99.02	3.219E-58			
basic helix-loop-helix family, member e40	*Bhlhe40*	99	4.795E-58			
nuclear receptor subfamily 1, group I, member 2	*Nr1i2*	99	4.795E-58			

Section 5. Genes encoding metabolic enzymes in the 99th percentile of the All nodes-Mm-liver transcriptomic consensome, deficiency of whose human orthologs is associated with a metabolic disorder.

Gene symbol links point to SPP transcriptomic Regulation Reports filtered for mouse liver. Disease links point to OMIM entries highlighted for the corresponding human gene.

Target	Gene product	CPV	Hepatic metabolic pathway	Known human deficiency disease
**Lipid metabolism**				
Ephx1	Epoxide hydroxylase 1, microsomal xenobiotic	7.27E-107	Conversion of epoxides to trans-dihydrodiols for conjugation and excretion	Familial hypercholanemia
Aldh3a2	Aldehyde dehydrogenase 3 family, member A2 (fatty aldehyde dehydrogenase)	2.11E-103	Rate-limiting oxidation of fatty aldehydes to fatty acids	Sjogren-Larsson syndrome
Ehhadh	Enoyl-Coenzyme A, hydratase/3-hydroxyacyl Coenzyme A dehydrogenase	4.70E-89	Essential for the production of medium-chain dicarboxylic acids	Fanconi renotubular syndrome 3
Hmgcs2	3-hydroxy-3-methylglutaryl-coenzyme A synthase 2, mitochondrial	1.23E-80	Rate-limiting enzyme of ketogenesis	HMG-CoA synthase-2 deficiency
Ephx2	Epoxide hydrolase 2, cytoplasmic	6.06E-78	Conversion of epoxides to trans-dihydrodiols for conjugation and excretion	Familial hypercholesterolemia
Decr1	2,4-dienoyl-CoA reductase 1	6.23E-71	Rate-limiting step of unsaturated fatty acid oxidation in mitochondria	DECR deficiency
Acadm	Acyl-Coenzyme A dehydrogenase, C-4 to C-12 straight chain	9.52E-65	Rate limiting step of medium-chain fatty acid β-oxidation	ACADM deficiency
Lpin2	Lipin 2	1.22E-65	Conversion of phosphatidic acid to diacylglycerol during triglyceride biosynthesis	Majeed syndrome
Lpl	Lipoprotein lipase	1.44E-62	Rate-limiting hydrolysis of core TGs from TG-rich lipoproteins	Familial combined hyperlipidemia
Acox1	Acyl-Coenzyme A oxidase 1, palmitoyl	3.53E-61	Rate-limiting enzyme in the peroxisomal fatty acid β-oxidation pathway	Peroxisomal acyl-CoA oxidase deficiency

Cpt2	Carnitine palmitoyltransferase II	3.22E-58	Rate limiting step in mitochondrial fatty acid beta-oxidation	Neonatal CPT II deficiency
Mlycd	Malonyl-CoA decarboxylase	1.44E-58	Conversion of malonyl-CoA to acetyl-CoA and CO_2	MLYCD deficiency

Carbohydrate metabolism

Pklr	pyruvate kinase L/R	1.04E-79	Rate-limiting step in glycolysis	Pyruvate kinase deficiency
Got1	Aspartate aminotransferase, cytoplasmic	4.30E-76	Transamination of aspartate & oxaloacetate in gluconeogenesis	Aspartate aminotransferase, serum level of, QTL1
Galk1	Galactokinase-1	2.66E-63	Phosphorylation of α-D-galactose to galactose 1-phosphate in the Leloir pathway	Galactokinase deficiency with cataracts
Aco2	Aconitase, mitochondrial	2.93E-59	Stereo-specific isomerization of citrate to isocitrate	Infantile cerebellar-retinal degeneration
Idh1	Isocitrate dehydrogenase, soluble	3.21E-58	Rate-limiting step of the TCA cycle	Glass syndrome, Ollier disease
Khk	Ketohexokinase (fructokinase)	3.21E-58	Rate-limiting conversion of fructose to fructose-1-phosphate	Fructosuria

Amino acid metabolism

Prodh	Proline dehydrogenase (proline oxidase)	2.54E-94	Rate-limiting oxidation of proline to glutamate	Hyperprolinemia
Hal	Histidine ammonia-lyase	3.55E-86	Initial reaction in histidine catabolism	Histidinemia
Asl	Argininosuccinate lyase	1.54E-83	Rate-limiting step in arginine synthesis	Argininosuccinic aciduria
Sardh	Sarcosine dehydrogenase	6.41E-67	N-demethylation of sarcosine to glycine	Sarcosinemia
Dmgdh	Dimethylglycine dehydrogenase	1.22E-65	Rate-limiting step in betaine metabolism	DMGDH deficiency
Aass	Alpha-aminoadipic semialdehyde synthase	3.09E-62	Rate-limiting step in lysine catabolism	Hyperlysinemia
Pah	Phenylalanine hydroxylase	3.09E-62	Rate-limiting step in phenylalanine catabolism	Phenylketonuria
Tat	Tyrosine aminotransferase, soluble	7.14E-62	Rate-limiting step in tyrosine catabolism	Type II tyrosinemia
Cth	Cystathionine gamma-lyase	9.20E-59	Rate-limiting step in cysteine synthesis	Cystathioninuria
Oat	Ornithine aminotransferase	3.14E-58	Glutamate biosynthesis	Gyrate atrophy

Other metabolic pathways

Cbs	Cystathionine beta-synthase	6.47E-87	Rate-limiting and initial enzyme in the trans-sulfuration pathway.	Homocysteinemia
Hsd3b2	3beta-hydroxysteroid dehydrogenase/delta(5)-delta(4)isomerase type II	1.01E-68	Rate limiting in aldosterone production	Congenital adrenal hyperplasia
Suox	Sulfite oxidase	2.12E-67	Oxidation of sulfite to sulfate	Sulfite oxidase deficiency
Gamt	Guanidinoacetate N-methyltransferase	1.22E-65	Creatine biosynthesis	Cerebral creatine deficiency
Comt	Catechol-O-methyltransferase	5.26E-65	Degradation of catecholamines	Panic disorder, schizophrenia
Alas2	Aminolevulinate, delta-, synthase-2	8.68E-63	Rate-limiting in heme biosynthesis	Sideroblastic anemia
Alpl	Alkaline phosphatase, liver/bone/kidney	1.86E-60	General hydrolysis of phosphate esters	Adult hypophosphatasia
Tgm1	Transglutaminase 1	1.08E-61	Rate limiting in formation of cross-linked protein envelope during terminal skin differentiation	Autosomal recessive congenital ichthyosis
Xdh	Xanthine dehydrogenase (xanthine oxidase)	2.33E-61	Rate-limiting enzyme in purine metabolism	Type I xanthinuria
Hmox1	Heme oxygenase 1	2.93E-59	Rate-limiting enzyme of heme degradation	HMOX1 deficiency
Cps1	Carbamoyl-phosphate synthetase 1, mitochondrial	3.14E-58	First rate-limiting mitochondrial enzyme in the urea cycle	CPS1 deficiency
Hsd17b10	17-beta-hydroxysteroid dehydrogenase X	3.21E-58	Beta-oxidation at position 17 of androgens and estrogens	HSD10 mitochondrial disease
Rdh5	Retinol dehydrogenase-5	4.39E-59	Rate limiting step in all-trans retinoic acid synthesis	Fundus albipunctatus

Section 6. Bench validation use case 2: ERR family members and insulin receptor regulate targets encoding glycogen synthase phosphatase and kinase regulatory subunits

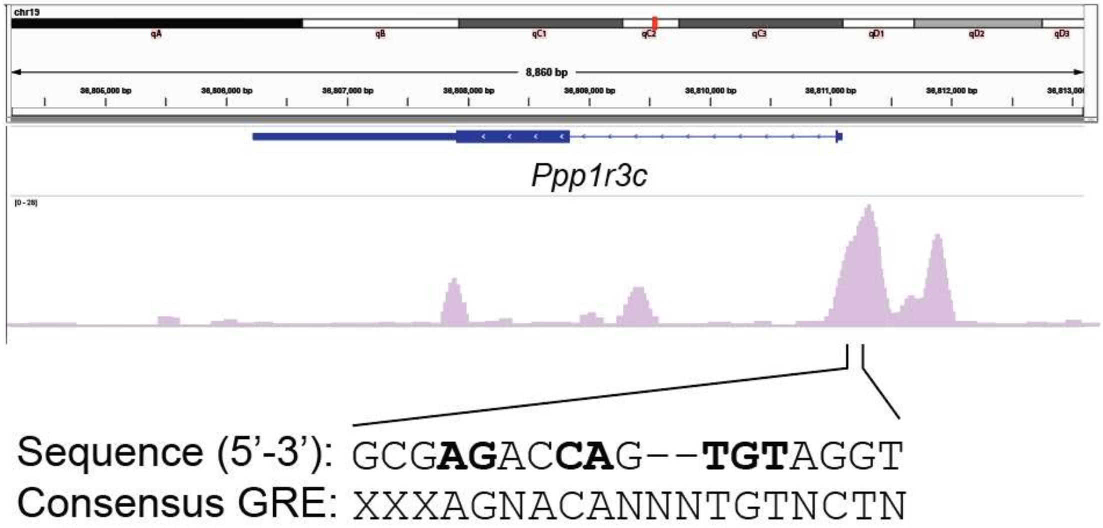

A. GR/NR3C1 ChIPseq data from inguinal adipose tissue (iWAT) showing two prominent peaks 5' to the first exon of PPP1R3C (gene is transcribed right to left in this image) and potential GRE within the first GR/NR3C1 peak. Based upon Ominer Regulation Report evidence for binding of GR to the *Ppp1r3c* promoter in mouse liver, we undertook sequence analysis of the murine *Ppp1r3c* promoter and identified two prominent peaks 5' to the first exon of *Ppp1r3c*, the more proximal of which contained a potential glucocorticoid response element (GRE, Fig. 5B; based on the GRE consensus {Reddy, 2009 #131}).

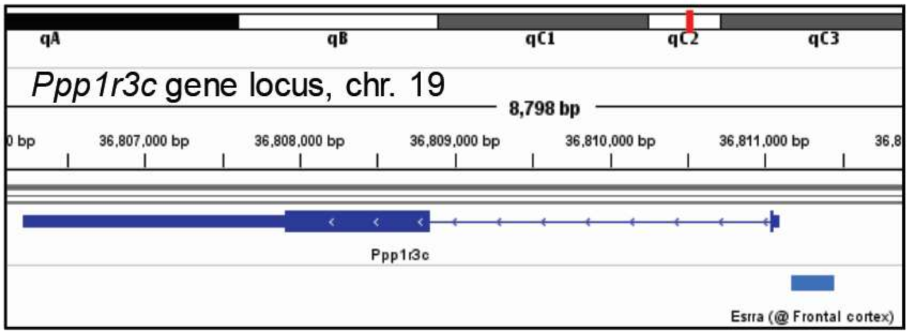

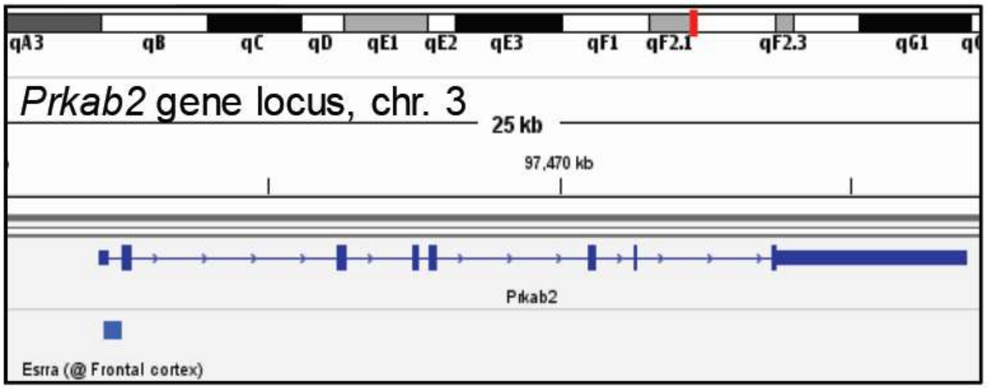

B. Evidence in the SPP cistromic Regulation Reports for *Ppp1r3c* and *Prkab2* and from IVG analysis of additional datasets supports the presence of one or more Esrra binding sites within 10 kb of the *Ppp1r3c* (upper) and *Prkab2* (lower) TSSs.

Section 7. Bench validation use case 3: the murine ERR, PPARGC and adipose tissue consensomes implicate *Mcrip2* in adipocyte oxidative metabolism

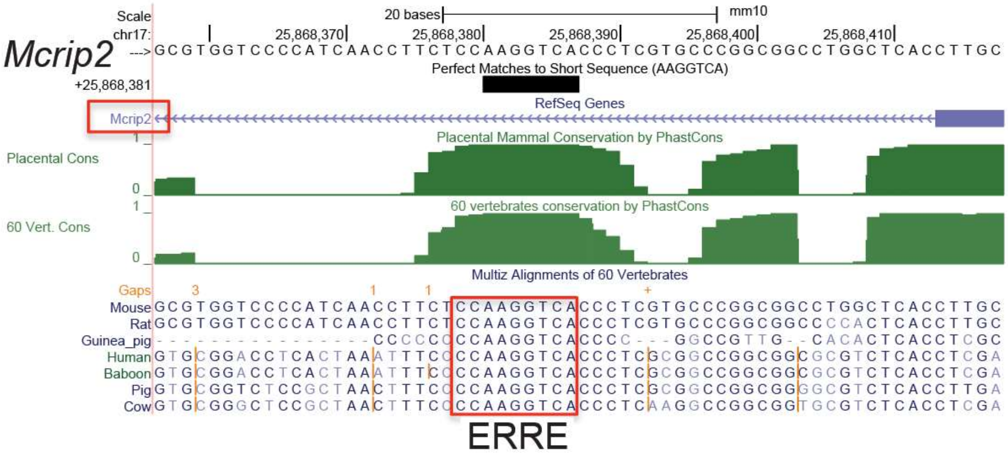

A. Putative ERREs in Mcrip2 locus. (source: UCSC genome browser).

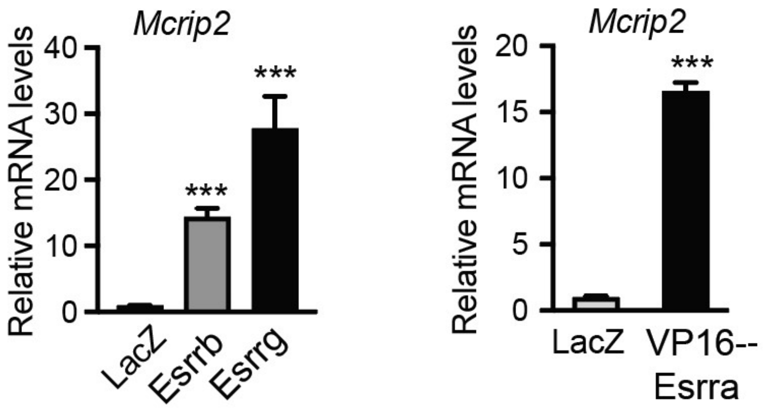

B. *Mcrip2* is induced by ERRs. C2C12 myotubes were infected with adenoviral vectors expressing LacZ, VP16-Esrra, Esrrb or Esrrg. RNA and data analyses are described in the Methods section.

Section 8. Transcriptomic consensome calculation for a hypothetical target

<table>
<tr><th>Gene</th><th>Experiment</th><th>Probeset</th><th>Fp</th><th>Pp</th><th>Zp</th><th>Fge</th><th>Pge</th><th>Eg</th><th>Ng</th><th>DR</th><th>CPV</th><th>GMFC</th></tr>
<tr><td>g</td><td>E1</td><td>PX1.1</td><td>4</td><td>0.001</td><td>3.29</td><td rowspan="3">2.0</td><td rowspan="3">0.08</td><td rowspan="6">2</td><td rowspan="6">4</td><td rowspan="6">0.5</td><td rowspan="6">0.014</td><td rowspan="6">2.83</td></tr>
<tr><td></td><td></td><td>PX1.2</td><td>2</td><td>0.05</td><td>1.96</td></tr>
<tr><td></td><td></td><td>PX1.3</td><td>1</td><td>1.0</td><td>0</td></tr>
<tr><td></td><td>E2</td><td>PX2</td><td>2.5</td><td>0.1</td><td>1.645</td><td>2.5</td><td>0.1</td></tr>
<tr><td></td><td>E3</td><td>PX3</td><td>0.333</td><td>0.02</td><td>-2.33</td><td>0.333</td><td>0.02</td></tr>
<tr><td></td><td>E4</td><td>PX4</td><td>0.333</td><td>0.02</td><td>-2.33</td><td>0.333</td><td>0.02</td></tr>
</table>

Section 9. Q-PCR primers for consensome validation

ERs-Hs-mammary gland transcriptomic consensome	
36B4	F: 5’-GGACATGTTGCTGGCCAATAA<br>R: 5’-GGGCCCGAGACCAGTGTT
ESR1	F: 5’-TGGAGATCTTCGACATGCTG<br>R: 5’-TCCAGAGACTTCAGGGTGCT
GREB1	F: 5’-TGGAAGGCTTGCAGCTCTTGAG<br>R: 5’-GGAAGGGCCGTGTAGCCTTCG
TPD52L1	F: 5’-ACTCGGCATGAACCTGATGA<br>R: 5’-CTGCGTGACTCAGGGTTTCA
CXCL12	F: 5’-ATTCTCAACACTCCAAACTGTGC<br>R: 5’-CTTCAGCCGGGCTACAATCTG
MYBL1	F: 5’-GGCGAAGAGGTCGCGCAGTG<br>R: 5’-TGCCATCGATGCTGGCACTGAA
FHL2	F: 5’-ATCCAAGTGCCAGGAATGCA<br>R: 5’-GTGGCAGATGAAGCAGGTCT
RAB31	F: 5’-CCATCGCTGGAAACAAGTGC<br>R: 5’-AACCACGATGGCACCTATGG
NPY1R	F: 5’-CCACTCTCCTCTTGGTGCTG<br>R: 5’-TGGTTTCACTGGACCTGTACT
IL17RB	F: 5’-GCCCTTCCATGTCTGTGAAT<br>R: 5’-ACTGAAGCTCGCGTTTGTTT
CA12	F: 5’-GTGCTCCTGCTGGTGATCTT<br>R: 5’-TGGACCAGCTATTCTCCCCA
NRIP1	F: 5’-CCAGCCCAAAATGAAGGTGC

	R: 5’-GTTTGCTGGGTCTCTGCTCT
MYC	F: 5’-CTACCCTCTCAACGACAG<br>R: 5’-TTCTTCCTCATCTTCTTGTTC
PRSS23	F: 5’-AAACCCACTTGGCCTGCATA<br>R: 5’-GGATGTAGATGCCCACCTGC
TFF1	F: 5’-TCCCCTGGTGCTTCTATCCTAATAC<br>R: 5’-GCAGTCAATCTGTGTTGTGAGCC
STC2	F: 5’-GACCGACGCCACCAACCCAC<br>R: 5’-CCCCACATCGCCAGCGTTGA
SLC7A5	F: 5’-GCCTACTTCACCACCCTGTC<br>R: 5’-AAGACGGGGATGATCCAGGA
EGR3	F: 5’-CATGTGCGGCGTGGAGTC<br>R: 5’-TAGGTCACGGTCTTGTTGCC
PTGES	F: 5’-CAGTATTGCAGGAGCGACCC<br>R: 5’-GACGAAGCCCAGGAAAAGGA
GR-mouse-liver transcriptomic consensome	
Ppp1r3b	F: 5’-TGAGCCACAGATTGCTGGAG<br>R: 5’-TGGATGTCCACAGCCATCAC
Ppp1r3c	F: 5’-TGATCCATGTGCTAGATCCACG<br>R: 5’-ACTCTGCGATTTGGCTTCCTG
Pcx	F: 5’-GGCTGCAGCAAGTTTGGTTG<br>R: 5’-TAGATGTTAGCTCCGCCCTG
Fgf21	F: 5’-AGCATACCCCATCCCTGACT<br>R: 5’-AAGAGACTTTCTGGACTGCG
Rplp0	F: 5’-GAAACTGCTGCCTCACATCCG<br>R: 5’-GCTGGCACAGTGACCTCACACG
